# Periodic radio and X-ray emission from an accreting white dwarf binary

**DOI:** 10.1038/s41550-026-02882-x

**Published:** 2026-06-01

**Authors:** Kovi Rose, Joshua Pritchard, Tara Murphy, L. N. Driessen, D. L. Kaplan, M. Caleb, Ziteng Wang, A. Zic, I. Andreoni, J. Carney, B. N. Barlow, D. Dobie, M. Gu, G. Heald, D. Huber, E. Lenc, J. K. Leung, W. Lu, R. Momose, M. G. Pedersen, Y. Qu, N. Rea, I. de Ruiter, K. Shaji, G. R. Sivakoff, A. J. M. Thomson, Y. L. Wang, G. J. Yang, F. Zahedy

**Affiliations:** 1 Sydney Institute for Astronomy, School of Physics, The University of Sydney, Sydney, New South Wales Australia; 2 Australia Telescope National Facility, CSIRO, Space & Astronomy, Epping, New South Wales Australia; 3 ARC Centre of Excellence for Gravitational Wave Discovery (OzGrav), Hawthorn, Victoria Australia; 4 Center for Gravitation, Cosmology, and Astrophysics, Department of Physics and Astronomy, University of Wisconsin-Milwaukee, Milwaukee, WI USA; 5 International Centre for Radio Astronomy Research, Curtin University, Bentley, Western Australia Australia; 6 Department of Physics and Astronomy, University of North Carolina, Chapel Hill, NC USA; 7 Department of Astronomy, Tsinghua University, Beijing, China; 8 The Hong Kong Institute for Astronomy and Astrophysics, The University of Hong Kong, Hong Kong, China; 9 SKA Observatory, SKA-Low Science Operations Centre, Kensington, Western Australia Australia; 10 Australia Telescope National Facility, CSIRO, Space & Astronomy, Kensington, Western Australia Australia; 11 Institute for Astronomy, University of Hawai’i, Honolulu, HI USA; 12 David A. Dunlap Department of Astronomy and Astrophysics, University of Toronto, Toronto, Ontario Canada; 13 Dunlap Institute for Astronomy and Astrophysics, University of Toronto, Toronto, Ontario Canada; 14 Racah Institute of Physics, The Hebrew University of Jerusalem, Jerusalem, Israel; 15 Department of Astronomy and Theoretical Astrophysics Center, University of California at Berkeley, Berkeley, CA USA; 16 Carnegie Science Observatories, Pasadena, CA USA; 17 Department of Astronomy, School of Science, The University of Tokyo, Bunkyo-ku, Japan; 18 Nevada Center for Astrophysics, University of Nevada, Las Vegas, NV USA; 19 Department of Physics and Astronomy, University of Nevada Las Vegas, Las Vegas, NV USA; 20 Institute of Space Sciences, ICE-CSIC, Barcelona, Spain; 21 Institut d’Estudis Espacials de Catalunya, Castelldefels, Spain; 22 Department of Physics, University of Alberta, Edmonton, Alberta Canada; 23 National Astronomical Observatories, Chinese Academy of Sciences, Beijing, China; 24 School of Astronomy and Space Science, University of Chinese Academy of Sciences, Beijing, China; 25 Department of Physics, University of North Texas, Denton, TX USA

**Keywords:** Stellar evolution, Stars, Transient astrophysical phenomena, Compact astrophysical objects

## Abstract

Long-period radio transients (LPTs) are coherent bursts of polarized radio emission that repeat periodically on timescales of minutes to hours. Little is known about the physical origins of these systems. Astronomers have proposed magnetars that rotate slowly and white dwarfs that rapidly orbit with a companion star as potential explanations. While several recent examples appear to support the latter hypothesis, the mechanism generating these bright radio pulses remains poorly understood. Here we report our discovery and classification of the LPT ASKAP J174508.9-505149 as an accreting white dwarf binary. This object has an ~1.3 h spectroscopic orbital period and exhibits orbitally modulated X-ray emission and radio bursts. These elliptically polarized radio bursts drift in emission frequency, potentially due to a longer beat period, and turn off for several hours at a time. Some LPTs have been associated with non-interacting white dwarf binaries. We have spectroscopically confirmed this system as an accreting cataclysmic variable, identified through characteristic optical emission lines and an ongoing X-ray outburst. Our results strengthen the link between at least some LPTs and white dwarf binaries.

## Abstract (web summary)

ASKAP J174508.9-505149 is a spectroscopically confirmed accreting white dwarf binary also identified as a long-period radio transient, strengthening the link between these mysterious bursting sources and white dwarf star systems.

## Main

We discovered ASKAP J174508.9-505149 (hereafter ASKAP J1745-5051) with the Australian SKA Pathfinder radio telescope (ASKAP)^1^ in an untargeted search for circularly polarized sources in the 1.365-GHz Rapid ASKAP Continuum Survey (RACS-mid)^2^ (Methods). In follow-up observations with the MeerKAT^3^ radio telescope (Methods) we refined the initial RACS-mid J2000 position to a right ascension (RA) of 17 h 45 min 8.929 ± 0.06 s and declination (dec.) −50° 51′ 49.86″ ± 0.03″.

**Fig. 1:**
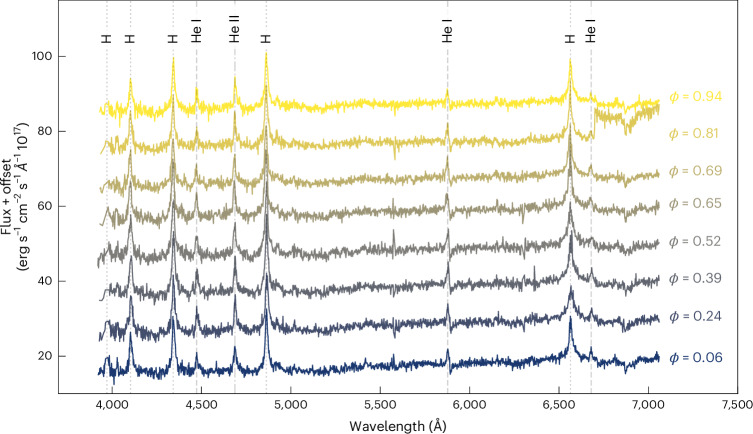
SOAR spectra of Gaia 4032. We show each of the consecutive 10-min spectra with an offset and plot the rest wavelengths for the hydrogen Balmer series (dotted), helium I (dashed) and helium II emission lines (dot-dashed). The orbital phases *ϕ* are shown next to each corresponding spectrum. The red excess in the *ϕ* = 0.81 spectrum (second from the top) is likely due to a calibration error.

**Extended Data Fig. 1:**
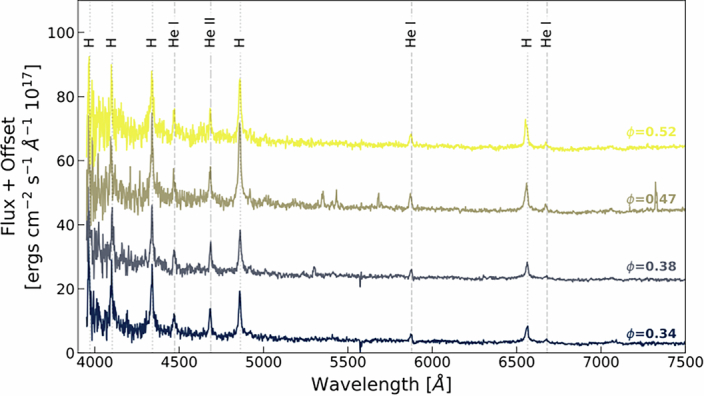
LDSS3 spectra of Gaia 4032. We show each of the 10 min spectra from both observing nights with an offset and plot the rest wavelengths for the Hydrogen Balmer series (dotted), Helium I (dashed), and Helium II emission lines (dot-dashed). The orbital phases *ϕ* are shown next to each corresponding spectrum.

We identified an optical counterpart in Gaia Data Release 3 (Gaia DR3)^4^, with an apparent magnitude of *m*_G_ = 19.45 ± 0.04 mag (Methods). In follow-up spectroscopy with the Southern Astrophysical Research (SOAR)/Goodman^5^ and Low Dispersion Survey Spectrograph (LDSS-3)/Magellan^6^ telescopes (Methods) we found ASKAP J1745-5051 to have a flat spectrum with a blue excess and strong, narrow emission features in Hydrogen (Balmer) and Helium (HeI, HeII) (Fig. 1 and Extended Data Fig. 1). The combination of strong HeII lines and the flat spectra with narrow Balmer lines are characteristic of magnetic cataclysmic variables (CVs) (for example, refs. ^7,8^). Magnetic CVs are compact binary systems composed of a strongly magnetized white dwarf and a main-sequence companion (usually of spectral type K to M)^9^, with polars and intermediate polars being the two main subtypes. Polars have close orbits (*P*_orb_ ≈ 1.3–4 h), and strong magnetic fields (*B* ≳ 10^7^ G) which synchronize the white dwarf spin to the orbital period^9^. Intermediate polars tend to have weaker magnetic fields (10^6^ ≲ *B* ≲ 10^7^ G) such that the white dwarf spin and orbital periods (*P*_orb_ ≈ 1.3–12 h) are not synchronized^9,10^. Polars may also deviate from synchronization for periods of ~100–1,000 years following a nova outburst^11^. White dwarfs in these asynchronous polars usually have spin periods a few per cent faster than *P*_orb_, but some systems, like Paloma (RX J0524+42), have been observed with spin periods up to ~20% faster than their orbital periods (see refs. ^12,13^, and references therein).

Another unique radio-emitting CV is AR Scorpii (AR Sco)^14^, which is more radio-luminous than most CVs^14^ and has been suggested as an evolutionary progenitor to intermediate polars and long-period radio transients (LPTs)^14,15^, although some have argued against this interpretation^16^. Like ASKAP J1745-5051, AR Sco also has flat optical spectra with narrow hydrogen and helium lines. We note that similar features are also seen in the two other known AR Sco-like systems J191213.72-441045.1 (J1912)^17^ and SDSS J230641.47+244055.8 (SDSS J2306)^16^. All three of these systems have orbital periods between 3.4 h and 4.1 h. Measuring Balmer line radial velocities (Methods), we found that ASKAP J1745-5051 has a far shorter orbital period of *P*_orb_ = 1.368 ± 0.053 h. This period is also shorter than ILT J1101+5521 (ILT J1101, *P*_orb_ = 2.1 h)^18^ and GLEAM-X J0704-37 (GLEAM-X J0740, *P*_orb_ = 2.9 h)^19^, LPTs thought to be associated with white dwarf-M dwarf binaries but lacking the characteristic spectra of a magnetic CV^20^. ASKAP J1745-5051 has properties broadly consistent with LPTs, namely, coherent and highly polarized radio bursts that repeat periodically. The observed LPT-like radio emission and magnetic CV-like spectral features of ASKAP J1745-5051 confirm this relationship and suggest that magnetic CVs may be the progenitor for a subset of LPTs.

**Fig. 2:**
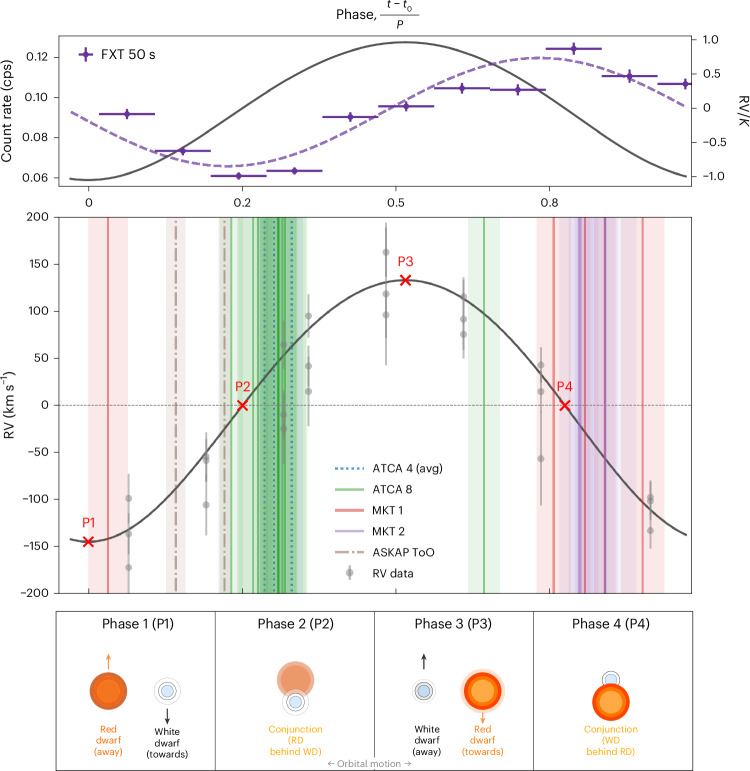
Phase-folded pulse timing. Top: Einstein Probe-FXT X-ray data compared with the normalized median two-body radial velocity posterior from The Joker (black curve), with the standard error of the binned count rates shown as vertical error bars and the width of the phase bins shown as horizontal error bars. We also show the sinusoid fitted to the X-ray data (dashed purple curve) peaking at an orbital phase of *ϕ*_X_ = 0.89 ± 0.19. Middle: arrival times of radio pulses compared with SOAR radial velocity measurements (grey markers with 1*σ* error bars) and median two-body radial velocity posterior from The Joker (black curve). We show pulses from MKT Epoch 1, MKT Epoch 2, and ATCA Epoch 8 in red, purple, and green, respectively. The ASKAP ToO pulses are denoted as dot-dashed brown lines. For the double-peaked pulses of ATCA Epoch 4 we show, in blue, the average pulse arrival times (dotted lines) taken halfway between the two peaks of each pulse. The light shaded regions denote the pulse width. The red crosses denote the binary phases shown below. Bottom: sketch of orbital phases with a face-on inclination. Phases 1 and 3 correspond to the binary quadratures—with the two stars side by side—where the Doppler shift maxima/minima occur. Phases 2 and 4 correspond to the binary conjunctions of the red dwarf (RD) and white dwarf (WD), when the radial velocity is zero.

Roughly 50 CVs have been seen to produce radio emission, including non-magnetic CVs (*B* ≲ 10^6^ G)^21–24^. Of these, none has been reported to exhibit periodic radio emission, and the observed radio emission in these CVs is far less luminous than that seen in LPTs, by a factor of at least 100–1,000. There have, however, been detections of coherent and highly circularly polarized radio emission from several magnetic CVs^23^ and one nova-like CV^21^, supporting a possible CV origin for LPTs. It has been shown that there is a canonical *P*_orb_ ≈ 1.3 h lower limit on CV orbital periods^25^, at which the white dwarf and its low-mass companion detach and begin to drift apart^26^. ASKAP J1745-5051 falls near this boundary, with an orbital period of *P*_orb_ = 1.368 ± 0.053 h. This spectroscopic period is consistent with the radio pulse period ${P}_{{\rm{radio}}}=1.3449{7}_{-0.00004}^{+0.00003}\,{\rm{h}}$, obtained from observations with the Australia Telescope Compact Array (ATCA)^27^ and ASKAP radio telescopes spanning nearly 2 years (Extended Data Table 1 and Methods). Moreover, phase-folding the arrival times of the radio bursts from separate observations revealed that these bursts occur around the same orbital phase near conjunctions, which occur at phases *ϕ* = 0.25, 0.75, with a median phase *ϕ*_median_ = 0.31 ± 0.03 for the ATCA and ASKAP bursts and *ϕ*_median_ = 0.8 ± 0.1 for the MeerKAT bursts (Fig. 2). Similar behaviour was observed from both AR Sco and ILT J1101, with radio lightcurves that peak around orbital conjunction^18,28,29^. It is noteworthy that, in Fig. 2, we see that the MeerKAT radio bursts are half an orbit out of phase with respect to the ASKAP and ATCA bursts, despite observing the complete orbital phase, indicating that there may be emission at both orbital conjunctions. We find no evidence for a seconds-long white dwarf spin period (Methods) similar to the seconds-long radio pulse structure seen in both AR Sco and ILT J1101^14,18^, and cannot directly constrain a white dwarf spin period on longer timescales.

**Fig. 3:**
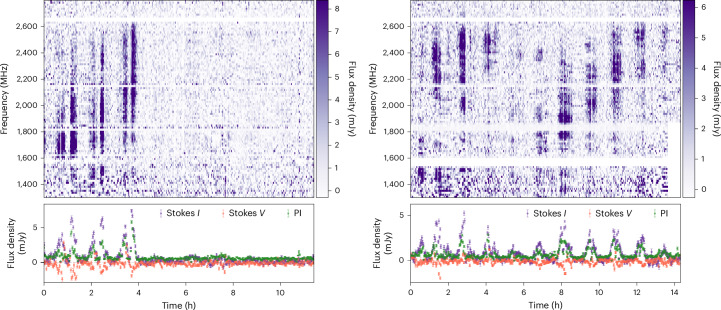
Total intensity (Stokes *I*) dynamic spectra of ASKAP J1745-5051 from ATCA. Corresponding Stokes *I*, *V* and polarized intensity ($\mathrm{PI}=\sqrt{{Q}^{2}+{U}^{2}}$) lightcurves are shown with 1*σ* standard error of the mean error bars. Both dynamic spectra, ATCA Epoch 4 (left) and ATCA Epoch 8 (right), use 15 MHz frequency averaging and 120 s time averaging. Empty regions of white space denote data that were flagged for radio frequency interference.

**Fig. 4:**
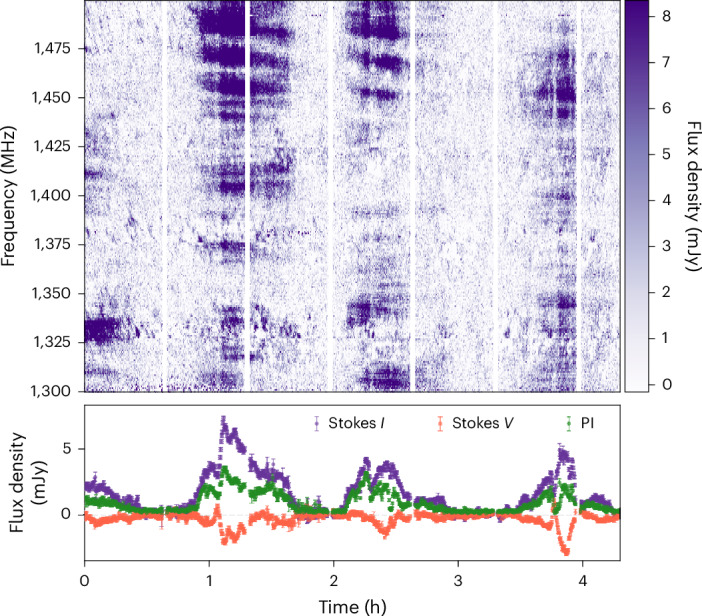
Total intensity (Stokes *I*) dynamic spectra of ASKAP J1745-5051 pulses from MKT Epoch 1. Corresponding Stokes *I*, *V* and $\mathrm{PI}=\sqrt{{Q}^{2}+{U}^{2}}$ lightcurves are shown with 1*σ* standard error of the mean error bars. These dynamic spectra (top) use 0.84 MHz frequency averaging and 16 s time averaging. We show the first half of the observation in the top of the observing band to highlight the modulation lane effect. Vertical white space corresponds to calibration scans.

**Extended Data Fig. 2:**
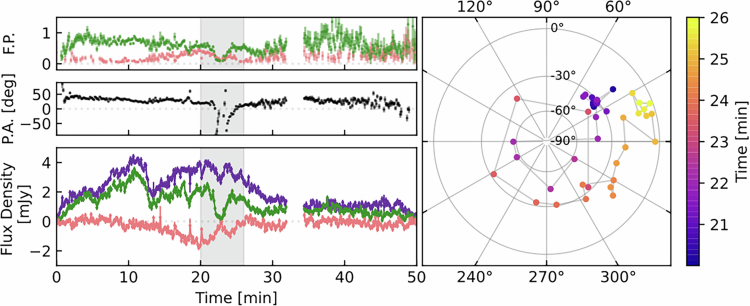
Polarization characteristics of a pulse detected in observation MKT Epoch 1. Left: The lower panel show the Stokes *I* (purple), Stokes *V* (pink), and PI (green) lightcurves. The middle panel shows the polarization position angle and the upper panel shows the circular (pink) and linear (green) polarization fractions. We use 1*σ* error bars in all panels. Right: A stereographic projection of the polarization state on the Poincaré sphere during the grey shaded region of the pulse, showing two complete revolutions about the Stokes *V* axis (-90°) before the polarization state returns to the original position angle.

The radio pulses from ASKAP J1745-5051 are elliptically polarized and display variability in their polarization properties (Extended Data Fig. 2 and Supplementary Data 1). ASKAP J1745-5051 also exhibits complex pulse morphology, narrowband emission structure and intermittency, including switching off for several hours at a time (Figs. 3 and 4).

ASKAP J1745-5051 exhibits pulse properties not previously observed in LPTs, providing valuable insights into the progenitor system. The pulses are seen to drift up and down in frequency over a longer beat period, with a modulation of the 2–3 GHz upper cut-off frequency (Fig. 3). ASKAP J1745-5051 also exhibits a narrow (~10 MHz) frequency structure within the pulses, shown in the MeerKAT dynamic spectra in Fig. 4. This sort of intensity modulation—commonly observed in the decametric emission from Jupiter^30^—is absent from all LPTs except for ASKAP J144834-685644 (ASKAP J1448)^31^. Such variability cannot be explained by interstellar propagation effects, with typical refractive interstellar scintillation having longer timescales (approximately months) and lower relative intensity variations (~10–30%), while diffractive interstellar scintillation would occur at much shorter timescales (~10 s) than we observe. This is the only time that these intensity patterns (also known as ‘modulation lanes’) have been detected in any binary system other than the Jupiter–Io system. The intensity modulation suggests the presence of local plasma acting as an interference screen to the beamed radio emission. The highly elliptical polarization and the radio frequency modulation indicate that the radio emission is coming from a strongly magnetized plasma.

**Extended Data Fig. 3:**
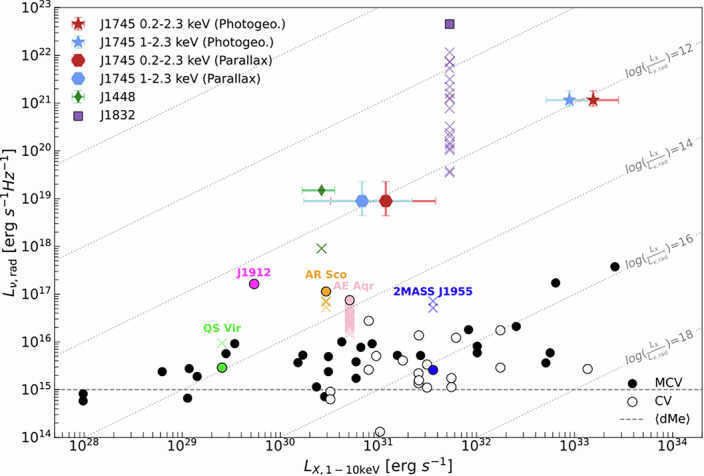
Radio and X-ray luminosities for CVs, MCVs, and LPTs with X-ray detections. The circles denote all CVs and confirmed white dwarf binaries^17,23,24^ with crosses showing coincident ASKAP measurements obtained from CASDA^90^. The horizontal dashed line denotes the mean flux for isolated M dwarfs. The radio luminosities for ASKAP J1745-5051, J1448 (diamond marker), and J1832 (square marker) are calculated with the maximum observed flux densities. We use the nominal 1 kpc distance for J1448 as indicative^31^. For ASKAP J1745-5051 we show both eROSITA bands assuming the median Bailer-Jones photogeometric distance (star markers), and the Gaia parallax distance (hexagon markers). We use the 1*σ* parallax uncertainties and the 16th and 84th percentiles of the Bailer-Jones distance posterior to calculate to error bars.

This plasma in the ASKAP J1745-5051 system may be the result of accretion onto the white dwarf. This is supported by the detection of coincident ultraviolet (UV) and X-ray emission in both archival observations and target-of-opportunity (ToO) observations we conducted with the Neil Gehrels Swift Observatory (Swift)^32^ and the Einstein Probe X-ray Telescope^33^ (Methods). We note that ASKAP J1745-5051 is only the third LPT detected at X-ray wavelengths, after the recent discoveries of ASKAP J1448 and ASKAP J1832-0911 (ASKAP J1832)^34^. AR Sco and J1912 also show pulsed X-ray emission^17,35^, the exact origin of which remains debated, although some residual accretion has been proposed for J1912^36^. Accretion in LPTs has been suggested only with the discovery of an X-ray outburst in ASKAP J1832^34^ but never proven unambiguously. The flux across the X-ray observations of ASKAP J1745-5051 varies by more than an order of magnitude, providing further evidence of variable accretion in the system. As seen with ASKAP J1832, we found that X-ray emission in ASKAP J1745-5051 varies periodically, at the same period as the radio pulsations, *P*_X_ = 1.32 ± 0.13 h (Fig. 2 and Methods). For ASKAP J1745-5051, this demonstrates that the X-ray periodicity is modulated by the orbital period and suggests that the same may be true for ASKAP J1832, with possible implications for the isolated neutron star or isolated white dwarf interpretations for ASKAP J1832. The X-ray emission is anti-phase with respect to ASKAP and ATCA but in phase with the MeerKAT radio bursts. Specifically, we find that the Einstein Probe data peak at an orbital phase of *ϕ*_X_ = 0.89 ± 0.19. This is consistent with the MeerKAT burst median phase and radial velocity posterior, but, with respect to the ATCA and ASKAP bursts, there is a phase delay of Δ*ϕ* = 0.58 ± 0.19 (Supplementary Information). The distance to ASKAP J1745-5051 is poorly constrained between 0.4 kpc and 9.1 kpc (Methods). We therefore calculate a limiting range of X-ray luminosities. We find that detections in the 0.2–10 keV band with luminosities *L*_X_ ≈ 10^30^–10^33^ erg s^−1^ are a good match for the typical range of accretion-generated X-ray emission in CVs^37^. Similarly, we constrain the RACS-mid radio luminosity *L*_R_ ≈ 10^18^–10^21^ erg s^−1^ Hz^−1^ at 1.365 GHz with a bandwidth of 288 MHz. This is more luminous than ~99% of all known radio stars^38^, making it unlikely that the radio emission originates from the stellar companion. We find that ASKAP J1745-5051 is also overluminous in the radio by a factor of ~100 (even at the lower distance limit) compared with all known CVs and most LPTs with both radio and X-ray detections (Extended Data Fig. 3). The notable LPT exception is ASKAP J1832, which has an estimated maximum radio luminosity of *L*_R_ ≈ 4 × 10^23^ erg s^−1^ Hz^−1^ (ref. ^34^).

**Extended Data Fig. 4:**
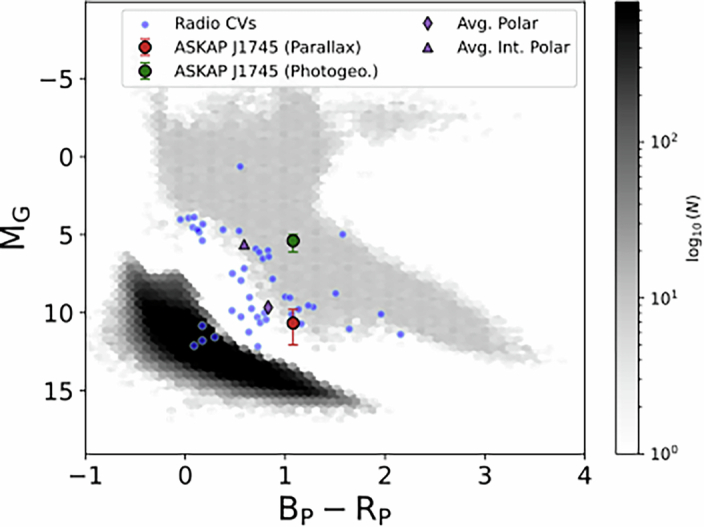
*Gaia* DR3 colour-magnitude diagram. We show radio-loud CVs with detected X-ray emission [^24^, and references therein] in blue and ASKAP J1745-5051; with the red marker denoting the value obtained from the Gaia DR3 parallax distance and the green marker denoting the value calculated with the median Bailer-Jones photogeometric distance. We use the 1*σ* parallax uncertainties and the 16th and 84th percentiles of the Bailer-Jones distance posterior to calculate to error bars. We also show the average Gaia centroids for polars and intermediate polars^93^ as a purple diamond and triangle, respectively. The background points show the Gaia the white dwarf population (black)^94^ as well as a sample of 10^7^ stars from Gaia DR3 (grey)^4^.

**Extended Data Fig. 5:**
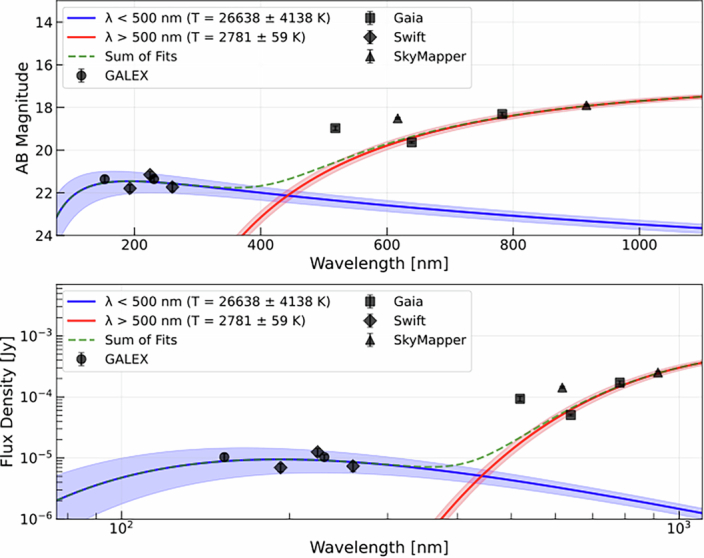
Blackbody temperature fits to photometric data. We show AB magnitudes (Top) and flux density (Bottom), both with 1*σ* errors. We fit the shorter wavelengths with *λ* < 500 nm (blue) and longer wavelength with *λ* < 500 nm separately (red), shown with 1*σ* uncertainty regions. We also show the sum of the fits as a dashed (green) line.

As evidenced by the optical spectra, ASKAP J1745-5051 appears to be a polar or asynchronous polar; however, without a constraint on the white dwarf spin period, we defer definitive classification to a future publication. Optical photometry and spectroscopy suggest a low-mass red or brown dwarf companion for ASKAP J1745-5051. Specifically, the apparent Gaia DR3 magnitude (*m*_G_ = 19.45 ± 0.04) is faint, and the spectra lack any obvious absorption lines or other spectral features. A white dwarf companion may be possible, but we consider this less likely as ASKAP J1745-5051 is redder and more luminous than most white dwarfs in the Gaia DR3 colour–magnitude diagram (Extended Data Fig. 4). Blackbody fits to the available photometry also suggest a low-mass spectral type M6.5 ± 0.5 companion (Methods and Extended Data Fig. 5), although these estimates may be contaminated by an unrelated nearby star and possibly by the accretion structure itself.

**Extended Data Fig. 6:**
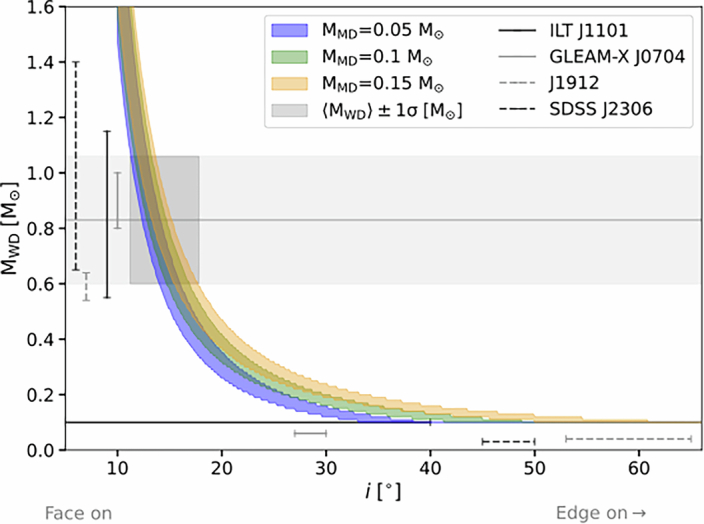
Binary mass functions for companion estimates. The grey line shows the empirical mean mass white dwarf for white dwarfs in a cataclysmic variable, within a ± 1*σ* region. The high-opacity subsection shows the range of inclinations (11 deg-17 deg) that would correspond to this white dwarf mass range for three test companion masses (0.05, 0.1, 0.15 *M*_⊙_). We show the inclination and mass ranges for similar systems for comparison.

Assuming the companion has filled its Roche lobe, which is the case for accreting CVs^9^, we can use the orbital period to estimate a companion mass and radius of the M dwarf: *M*_MD_ = 0.0963 ± 0.0047 masses of the Sun (*M*_⊙_) and *R*_MD_ = 0.1321 ± 0.0055 radii of the Sun (*R*_⊙_)^9^ (Methods). These values fall on the lower end of M dwarf values, corresponding to an ~M6 companion—in line with the blackbody spectral type. Taking the empirical mean mass for white dwarfs in a CV: *M*_WD_ = 0.83 ± 0.23 *M*_⊙_ (ref. ^39^), we obtain an orbital separation of *a* = 4.2 ± 0.4 × 10^10^ cm = 0.61 ± 0.05 *R*_⊙_. Using this white dwarf mass with the orbital period and radial velocity amplitude, the binary mass function for the estimated *M*_MD_ ≈ 0.10 *M*_⊙_ companion constrains the system inclination to *i* = 14 ± 3 deg (Extended Data Fig. 6 and Methods). We find that the system is highly inclined (face-on), regardless of the exact companion mass.

Low-mass M dwarfs and cooler, fully convective brown dwarfs can produce detectable radio emission (for example, refs. ^40,41^). These dwarfs possess surface magnetic fields of up to a few kilogauss, which are understood to be involved in the generation of this radio emission (for example, refs. ^42,43^)—with typical luminosities four orders of magnitude lower than ASKAP J1745-5051 at gigahertz frequencies^44^. While white dwarfs in CVs typically have much stronger surface magnetic fields (of order megagauss), the magnetic field strength at any emission site would depend on its location relative to the two objects in the binary. The detected emission from ASKAP J1745-5051—with a brightness temperature lower limit of *T*_B_ > 10^12^ K (Methods)—is necessarily produced by a coherent process, likely arising in the combined magnetic field interaction between the white dwarf and its companion.

For example, it has been suggested that the orbital motion of a weakly magnetized M dwarf within a strong white dwarf magnetosphere can produce a unipolar inductor effect^45^. As electrons from the accreted plasma are accelerated along the interacting magnetic field lines, both the background white dwarf field strength and the electron Lorentz factor grow. This can produce the observed *L*_X_ ≈ 10^30^–10^33^ erg s^−1^ X-ray emission from relativistically boosted cyclotron radiation. We note that accretion and inverse Compton scattering could also produce similar levels of X-ray emission^37^. Electron cyclotron maser emission (ECME) (for example, ref. ^46^) can plausibly be generated in low-density regions of the same accreting plasma, for example at higher altitudes of the accretion column between the two stars.

The coherent, circularly polarized emission from low-mass stars is widely thought to be generated by ECME^47^. However, the degree of linear polarization and high radio luminosity in the ASKAP J1745-5051 pulses are not typical of standard ECME or other emission mechanisms operating in typical stellar atmospheres (for example, ref. ^38^), making it unlikely that the emission originates solely from stellar magnetic activity of the M dwarf companion.

We suggest that a contribution from relativistic ECME—possibly due to the magnetospheric interaction^45,48^—may account for the high linear polarization and boost the radio luminosity. ASKAP J1745-5051 also exhibits rapid changes in polarization and swings in polarization position angle (PA) (Extended Data Fig. 2). This may be due to the precession of the emitting region relative to our line of sight^49^ and the interaction of the ECME beam with surrounding magnetospheric plasma. In the Jupiter–Io system, hollow-cone ECME is generated as the moon Io energizes particles along the field lines in Jupiter’s magnetosphere. This beamed decametric emission produces a thin-film interference pattern when it passes through local plasma^30^. In the case of ASKAP J1745-5051, we propose that similar plasma enhancements from accreted material may be responsible for the observed intensity modulations.

We see evidence of this plasma environment from Balmer emission lines, with an equivalent width ratio of H_α_/H_β_ ≤ 1 indicative of electron densities *n*_e_ ≳ 10^13^ cm^−3^ (see ref. ^50^, and references therein). This strength ratio varies over time but is, on average, consistent between observations, with median values of 0.66 ± 0.03 in SOAR (Fig. 1) and 0.68 ± 0.07 in LDSS-3 (Extended Data Fig. 1). We also see short-timescale variability in the HeII/H_β_ ratio, indicative of channelized accretion^8^. As with the Balmer lines, this equivalent width ratio is also consistent across observations, with median values of 0.415 ± 0.018 in SOAR and 0.39 ± 0.08 in LDSS-3. We tabulate the line strengths and ratios in Supplementary Tables 3 and 4. The variability in relative H_α_ emission indicates changes in the local electron density and may be suggestive of variable accretion. It may also be indicative of instabilities in an accretion disk.

**Extended Data Fig. 7:**
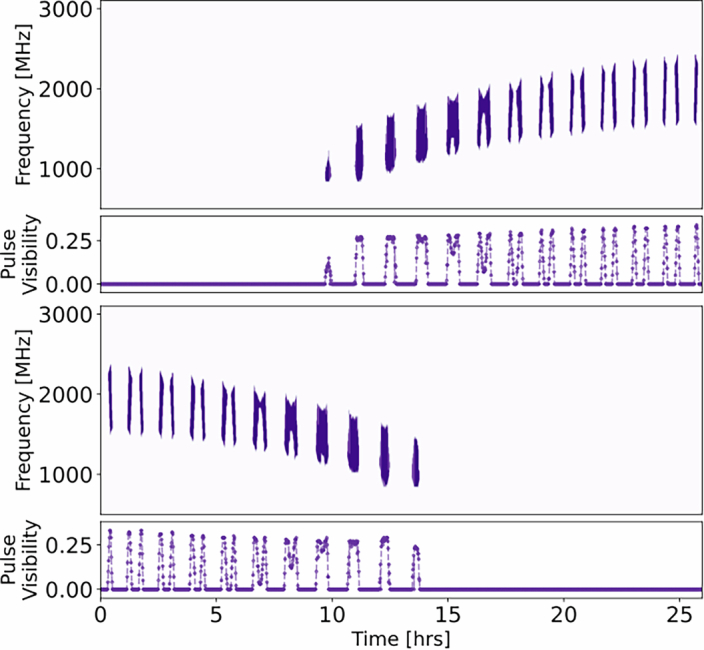
Example synthetic dynamic spectra generated from a geometric model representing ASKAP J1745-5051 as a pair of interacting magnetic dipoles in a near synchronous binary. Each panel shows the simulated visibility of ECME as a function of frequency and time over several orbital cycles, where non-zero values indicate the presence of at least one viable emission site with the appropriate local field strength and orientation. The rising (Top) and falling (Bottom) of the pulse frequency corresponds to different phases of the beat period. Lower panels show the simulations as a time series of emission visibility. The time axis begins at inferior white dwarf conjunction. The model reproduces several key features of radio pulses in our observations including: double-peaked pulses, variable spacing been pulse pairs, modulation of frequency cutoffs, and intermittency.

The intermittency and frequency cut-off modulation in ASKAP J1745-5051 could also be explained by asynchronous rotation of the white dwarf and an inclined magnetic axis, which may be the result of a past nova outburst in the system^11^. We find that a simple geometric model of dipolar magnetic fields in an asynchronous orbit, with strengths of order megagauss and kilogauss for the white dwarf and M dwarf, respectively, can reproduce both the variability and frequency evolution^48^ (see the Methods for details). In this model, the radio emission is produced in an interaction region. This is required for a magnetic CV, as emission from closer to the white dwarf surface would require a gyrofrequency an order of magnitude larger than the observed approximately gigahertz emission. In Extended Data Fig. 7, we show simulated dynamic spectra generated with this approach. While our model does not include the plasma physics and gravitational interaction relevant to accreting binaries, our model can reproduce the observed intermittency, radio frequency cut-off modulation and variable gap width between pulse pairs (Fig. 3). This interaction model may also be applicable to other LPTs with white dwarf binary progenitors.

Varying conditions in the local plasma density and magnetic field interaction may explain the intermittency and unique pulse morphologies in the observed radio pulsations from ASKAP J1745-5051. The fact that these pulsations from ASKAP J1745-5051 are mostly phase-aligned around conjunction shows similarity to AR Sco, which was found to produce orbitally modulated radio bursts around the same orbital phase—at or near conjunction^28^. Evidence of similar behaviour was found in ILT J1101, with spectroscopic analysis suggesting the LPT was associated with an M dwarf in a binary system, along with a blue photometric excess hinting at a white dwarf companion^18^. We note that this is not the case for GLEAM-X J0740; however, recent work suggests that this may be a geometry-dependent effect^51^.

Our observations of ASKAP J1745-5051 demonstrate that magnetically driven accretion plays a key role in the generation of emission across the electromagnetic spectrum in magnetic CVs, including coherent radio pulses and variable X-ray emission. The discovery of ASKAP J1745-5051, and its modulated emission in the radio and X-ray bands associated with the spectroscopic orbital period, clearly establishes that accreting CVs make up at least part of the population of LPTs. Future long-duration optical photometry and spectropolarimetry observations will help to constrain the properties of the low-mass companion. Coordinating these observations with simultaneous radio and X-ray observations will further establish the role that magnetically driven accretion plays in generating periodically pulsed emission in these systems. Determining if these processes can explain the properties of the entire emerging class of LPTs will require detailed simulations and modelling, as well as the discovery and investigation of new LPTs.

## Methods

## Observations

### ASKAP

Of the ~3 × 10^6^ sources detected in the RACS-mid survey, only ~100 are highly circularly polarized (polarization fraction >10%). Following the approach used for polarization searches of RACS^40,41^, we selected ASKAP J1745-5051 for further study as it was the only one of these 100 circularly polarized sources without a known astronomical identification within 10″.

ASKAP J1745-5051 was detected with a time- and frequency-averaged Stokes *I* flux density of 1.053 ± 0.085 mJy per beam at 943.5 MHz (SBID 63789), and was detected at 1.365 GHz with flux densities of 22.9 ± 2.3 mJy per beam (SBID 20398) and 2.51 ± 0.26 mJy per beam (SBID 76988). We obtain 5*σ* flux density limits—calculated as five times the root-mean-square noise—of 1.22 mJy per beam at 887.5 MHz (SBID 8646) and 0.53 mJy per beam at 855.5 MHz (SBID 34553). The continuum detections and limits from ASKAP are summarized in Extended Data Table 1.

### ATCA

We conducted follow-up radio observations of ASKAP J1745-5051 with ATCA^27^ (project codes C3363, CX553 and C3587). We obtained 97 h of L-band (1.1–3.1 GHz) and 6 h of simultaneous C-band (4.5–6.5 GHz) and X-band (8.0–10.0 GHz) observations, using the extended 6-km array configuration. The details of these observations—including shorthand names for each observation—are summarized in Extended Data Table 1.

We used standard continuum data reduction routines with MIRIAD^52^ to flag and calibrate the data. We used the ATCA primary calibrator source PKS B1934-638 to calibrate the bandpass response and flux scale for all observations except ATCA Epoch 6 and ATCA Epoch 7, for which we used PKS B0823-500. For all observations, we corrected time-varying gains using interleaved scans on the calibrator PKS 1740-517.

### Murriyang

We conducted a 2-h Director’s Discretionary Time observation with Murriyang, the CSIRO Parkes Radio Telescope. We used 32-μs time sampling and 1-MHz channel frequency resolution across the 26 × 128 MHz subbands in the 704–4,032 MHz range of the ultrawide-bandwidth, low-frequency receiver^53^.

### MeerKAT

We conducted follow-up radio observations of ASKAP J1745-5051 with the MeerKAT radio telescope^3^; project ID: SCI-20241101-KR-02. We obtained three 10-h observations with the L-band (856–1,712 MHz) receiver, using the c856M1k correlator configuration with an 8-s integration time. We used the SARAO Science Data Processor pipeline to flag and calibrate the data, using PKS J1939-6342 to calibrate the delays, bandpass response, flux scale and polarization leakage, PKS J1744-5144 to calibrate the time-varying gains, and PKS J1331+3030 to set the absolute polarization PA. We additionally corrected the visibilities for mislabelling of the X and Y feeds, which produce a sign inversion in Stokes *Q* and Stokes *V* and rotate the polarization angle by 90° if left uncorrected^54^.

### Archival radio searches

We searched the archives of the most sensitive radio, millimetre and submillimetre arrays that can observe a source at −50 declination. We did not identify archival radio observations covering the position ASKAP J1745-5051 from the Atacama Large Millimeter/submillimeter Array (ALMA)^55^, nor were there any observations made with ATCA, before this work, available on the Australia Telescope Online Archive (https://atoa.atnf.csiro.au/query.jsp). We identified ASKAP J1745-5051 in the MeerKAT Absorption Line Survey (MALS)^56^, conducted at 1.28 GHz with a bandwidth of 856 MHz. ASKAP J1745-5051 is active at 3–5*σ* levels in an observation starting on 2020-09-20 13:50 UTC. We provide some details on these nominal detections in the Supplementary Information.

### Gaia (including distance estimate)

In the third data release of the Gaia^57^ space-based optical telescope (Gaia DR3)^4^, we identify two faint sources within ~0.6″ of ASKAP J1745-5051, located at distances between approximately 0.4 kpc and 2.0 kpc, accounting for large parallax uncertainties. Both the original RACS-mid position and the average position across the ATCA observations are within ~0.3″ of Gaia 5946454415417964032 (henceforth Gaia 4032), with the MKT Epoch 1 position separated by 0.16″ from Gaia 4032. By comparison, the offsets from the other Gaia source—Gaia 5946454411127231488 (henceforth Gaia 1488)—are 0.61″ (RACS-mid), 0.90″ (MKT Epoch 1) and 0.85″ (ATCA median separation). This supports an association between ASKAP J1745-5051 and Gaia 4032.

Gaia 4032 is located in the Main Sequence of the Gaia colour–magnitude diagram (Extended Data Fig. 4), with an apparent magnitude of *m*_G_ = 19.40 ± 0.04 mag and colour index of *B*_P_ − *R*_P_ = 1.079. Gaia 4032 has a DR3 parallax of *ϖ* = 1.75 ± 0.91 mas, corresponding to a parallax distance of ${d}_{\varpi }=0.5{7}_{-0.19}^{+0.62}\,\mathrm{kpc}$ and an absolute Gaia magnitude ${M}_{\mathrm{G,parallax}}=10.6{7}_{-0.88}^{+1.39}\,\mathrm{mag}$, without correcting for extinction. This absolute magnitude is typical of an M dwarf ^58^, and the space velocity, calculated with the Gaia parallax and proper motions, is consistent with the Galactic average^59^. However, as the uncertainties on the Gaia parallax distance and proper motion are poorly constrained (Supplementary Information), and because our conclusions are not sensitive to the exact distance adopted, we choose not to assume a single preferred value.

Instead, we select a larger plausible distance range, including the ‘Bailer–Jones’ photogeometric distance estimate^60^, to ensure a conservative interpretation. The photogeometric distance is determined from Gaia parallax and photometry using a probabilistic approach. This method is considered more reliable for Gaia sources with fractional parallax uncertainties in the range 0.1 ≤ *σ*_*ϖ*_/*ϖ* ≤ 1 (ref. ^60^), as is the case for Gaia 4032. This catalogue provides the median photogeometric distance of ${d}_{\mathrm{photogeo}}=6.{5}_{-1.1}^{+2.6}\,\mathrm{kpc}$ for Gaia 4032. We note that the photogeometric distance may be skewed by emission from the companion and accretion, as it relies on photometric priors. The Bailer–Jones^60^ photogeometric distance corresponds to the absolute magnitude ${M}_{\mathrm{G,photogeo}}=5.3{9}_{-0.73}^{+0.40}\,\mathrm{mag}$, without correcting for extinction. Hence, combining the parallax and photogeometric distances, we adopt the distance range 0.4–9.1 kpc. We note that this range is extremely conservative, and that considerations of typical absolute magnitudes for M dwarfs suggest that the true distance is closer to the parallax distance. Regardless of the precise value, our interpretations of the system and emission mechanisms remain unaffected.

### LDSS-3

We conducted optical spectroscopic observations with the LDSS-3 spectrograph on the 6.5-m Magellan Clay telescope at the Las Campanas Observatory. We used the volume phase holographic (VPH)-All grism (4,250–10,000 Å) with a 1″ slit.

In the first observation, starting at 2024-03-07 07:36 UTC, the slit was aligned in an east–west configuration, and we obtained 2 × 600 s of data. With this slit alignment we obtained spectra for Gaia 4032 and Gaia 1488 simultaneously. We conducted a second observation starting at 2024-03-08 07:53 UTC, aligning the slit in a north–south configuration to observe each of the Gaia sources separately. We obtained 2 × 600 s exposures of Gaia 4032 and 4 × 600 s of Gaia 1488. We used a version of the reduction pipeline developed for the Magellan IMACS spectrograph^61^, with updated wavelength calibration and sky subtraction^62^. See the Supplementary Information for further details.

### SOAR

We took optical spectroscopic observations of ASKAP J1745-5051 with the Goodman High Throughput Spectrograph (GHTS)^5^ on the SOAR 4.1-m telescope. We obtained 8 × 600 s consecutive exposures (principal investigator: I.A.) starting between 2024-09-25 00:34 and 2024-09-25 01:52 UTC. Each observation covered a wavelength range of 3,800–7,050 Å and was conducted using a single 1″ slit mask (aligned with the parallactic angle) and a VPH grating with 400 lines mm^−1^.

The data were reduced with PypeIt^63^ using standard procedures for bias subtraction, flat-fielding, wavelength calibration and fluxing using observations of a bright calibration star.

### GALEX

We identified a nearby UV source, GALEX J174508.8-505149, separated from ASKAP J1745-5051 by 0.42″. GALEX is the Galaxy Evolution Explorer^64^ space telescope. This source has a far-UV (1,528 Å) magnitude of *M*_FUV_ = 19.84 ± 0.15 mag and a near-UV (2,310 Å) magnitude of *M*_NUV_ = 19.67 ± 0.11 mag, with the zero-point calibrated on the AB magnitude scale. These magnitudes correspond to the calibrated flux densities *F*_FUV_ = 42.1 ± 5.8 μJy and *F*_UV_ = 49.3 ± 4.8 μJy.

### eROSITA

We identified a nearby X-ray source, 1eRASS J174508.8-505151, detected by the Russian-German Spektr-RG (SRG) space-based telescope in the eROSITA (extended ROentgen Survey with an Imaging Telescope Array) all-sky survey (SRG/eRASS)^65^. In the 0.2–2.3 keV band, 1eRASS J174508.8-505151 is offset from ASKAP J1745-5051 by 1.2″ in both MKT Epoch 1 and Gaia DR3. In the 2.3–5.0 keV band, 1eRASS J174508.8-505151 is offset from ASKAP J1745-5051 by 0.2″ in both MKT Epoch 1 and Gaia DR3. The eROSITA flux is *F*_X,soft_ = 3.1 ± 0.6 × 10^−13^ erg s^−1^ cm^−2^ in the soft 0.2–2.3 keV, and *F*_X,hard_ = 1.7 ± 0.4 × 10^−13^ erg s^−1^ cm^−2^ in the hard 2.3–5.0 keV band.

### Swift

We did not find any archival detections of ASKAP J1745-5051 with the Neil Gehrels Swift Observatory (Swift^32^), a space telescope that performs simultaneous UV and X-ray photometry, within a 10′ cone search. We obtained a 1.1-ks Swift ToO observation (https://heasarc.gsfc.nasa.gov/FTP/swift/data/obs/2024_05//00016563005/), starting at 2024-05-16 17:43 UTC. We split this observation into three 0.36-ks exposures, with one exposure for each Swift UVOT band used here—UVW2, UVM2 and UVW1—which correspond to 1,928 Å, 2,246 Å and 2,600 Å. The Swift X-Ray Telescope (XRT) instrument observed simultaneously in PHOTONCOUNTING mode for the duration of the observation.

We reduced and analysed the Swift data on the SciServer online compute platform^66^ using the National Aeronautics and Space Administration (NASA)’s High Energy Astrophysics Science Archive Research Center (HEASARC) (https://heasarc.gsfc.nasa.gov/docs/software.html) software package. See the Supplementary Information for additional details. In observation ID 00016563005, we found a single source in all three UV bands, each within <0.8″ of the ASKAP J1745-5051 radio position (Supplementary Table 2).

We also inspected simultaneous data from Swift XRT to obtain a count of 0.3–10.0 keV photons in a 15″ aperture. We find an XRT count of 14.0 ± 3.7 photons in the observation (ID 00016563005), corresponding to a rate of $1.{3}_{-0.9}^{+1.6}\times 1{0}^{-2}$ counts s^−1^. We used the HEASARC Portable Interactive Multi-Mission Simulator (https://heasarc.gsfc.nasa.gov/cgi-bin/Tools/w3pimms/w3pimms.pl), with a power law photon index of 2, to predict 0.3–10.0 keV flux of ${F}_{{\rm{PIMSS}}}=4.{8}_{-3.6}^{+6.1}\times 1{0}^{-13}$ erg s^−1^ cm^−2^.

### Einstein Probe

We triggered ToO observations of ASKAP J1745-5051 with the Einstein Probe’s Follow-up X-ray Telescope (FXT). Three observations were respectively performed at 2025-09-13 18:04:13 UTC (9.2 ks exposure), 2025-09-19 13:27:05 UTC (10.5 ks) and 2025-09-23 19:42:47 UTC (10.0 ks). During the observations, both modules of FXT were set in Full Frame mode, which has a timing resolution of 50 ms. Spectral analysis was performed with XSPEC v12.14.1. We applied a tbabs × power law model to fit the X-ray spectra. The source flux in the 0.5–10 keV band showed a slow decreasing trend, from $2.7{5}_{-0.23}^{+0.25}\times 1{0}^{-12}$ erg s^−1^ cm^−2^ in the first FXT observation to $1.6{7}_{-0.15}^{+0.14}\times 1{0}^{-12}$ erg s^−1^ cm^−2^ in the last one. Within errors, the fitted values of the photon index are consistent in the three observations, varying from $1.2{2}_{-0.17}^{+0.19}$ to $1.2{8}_{-0.16}^{+0.19}$. The fitted values of *n*_H_ are also roughly consistent with the Galactic value. Further analysis of the X-ray emission from ASKAP J1745-5051 will be published in a follow-up paper.

## Analysis

### Chance coincidence

We conducted chance coincidence trials to quantify the likelihood of spatially matching ASKAP J1745-5051 to an unrelated multiwavelength source. For this, we used our best astrometric measurements, from MKT Epoch 1.

We produced catalogues of all sources within a search radius from ASKAP J1745-5051 of 0.2 deg for Gaia DR3, 1 deg for GALEX and 3 deg for eROSITA, respectively containing 21,982, 8,871 and 736 sources. We then ran *n* = 10^5^, trials shifting the position of ASKAP J1745-5051 by an angle selected randomly from a uniform distribution between 0° and 360° and a radial separation generated from a random uniform distribution ranging from 0.25′ to the maximum extent of the respective search radius. For each trial, we identify a match as being within 0.3″ (Gaia), 0.5″ (GALEX) and 11.25″ (eROSITA). These are based on 5*σ* regions defined by the respective astrometric uncertainties of MeerKAT, GALEX^67^ and eROSITA^65^, respectively.

From these random trials, we defined the chance coincidence probability as (*m* + 1)/(*n* + 2), where *m* is the total number of matches to these catalogues within the given cross-match radius. We found that the probability of finding a random source within the respective cross-match radius is 0.061% for eROSITA (with *m* = 61 matches), 0.022% for GALEX (with *m* = 21 matches) and 0.37% for Gaia (with *m* = 361 matches).

### Line fitting

We observed strong H_β_, H_γ_ and H_δ_ emission lines from Gaia 4032 in all LDSS-3 exposures (Extended Data Fig. 1). We do not observe any obvious emission features from Gaia 1488 in any observations. The narrow spectral feature around ~7,300 Å is probably the result of cosmic rays.

The SOAR spectra (Fig. 1) also show strong Balmer lines and He lines in emission, with no obvious absorption features. The narrow spectral features around ~5,600 Å and ~6,300 Å are probably residuals from the sky lines at 5,578.5 Å and 6,301.7 Å. The red excess in the penultimate spectrum (second from the top in Fig. 1) is likely due to a calibration error.

We fitted each of the high signal-to-noise emission lines in the SOAR and LDSS-3 spectra and calculated the radial velocity (RV) using *λ*_fit_. We calculated the equivalent width for each of these high signal-to-noise emission lines in both datasets. Supplementary Tables 3 and 4 contain values obtained for the equivalent widths, radial velocities and full width at half maximum for H_α_, H_β_, H_γ_, H_δ_ (only LDSS-3) and HeII.

We obtained the radial velocities for the three lines with the best signal-to-noise ratios in the SOAR spectra (H_α_, H_β_ and H_γ_) and input these data into The Joker Monte Carlo sampler of radial velocity curves for two-body systems^68^ to extract the binary orbital parameters. We generated 10^6^ samples and used the default prior distributions. In Supplementary Fig. 1, we show the posterior sample lightcurves with the RV values overplotted. A clear sinusoidal trend can be seen in the radial velocity over the ~1.3 h in which the spectra were taken. Specifically, we obtained a median period of *P*_orb_ = 1.369 ± 0.053 h as well as median velocities of *K* = 114.2 ± 7.5 km s^−1^ for the semi-amplitude and *v*_0_ = 15.8 ± 5.0 km s^−1^ for the barycentre velocity of the binary system.

These narrow emission lines with wide bases are typical of polar and intermediate polar systems^7^. Strong HeII lines are common in polars as well as longer-period CVs, with the 4,686 Å HeII line indicating a magnetic white dwarf in most CVs where it is detected^69^. When such magnetic systems have highly channelled accretion, the strength of the HeII emission tends to be comparable to that of the Balmer lines^8^. The combination of strong, narrow Balmer and HeII lines—usually seen in polars undergoing active accretion^7^—and the shorter ~1.3 h orbital period support the polar interpretation. While the optical spectra of polar and intermediate polars are often quite similar, the HeII in intermediate polars is usually weaker than the H_β_ (ref. ^9^). The HeII/H_β_ line ratios from SOAR and LDSS-3 (Supplementary Tables 3 and 4) might therefore suggest that ASKAP J1745-5051 is an intermediate polar.

### Physical properties

We use two independent approaches to estimate the mass of the companion *M*_MD_. The first assumes that the companion has filled its Roche lobe and relies on the relationship between the mean density of the companion and the orbital period of the system^9^. These provide the mean empirical mass–period and radius–period relationships

1 $${M}_{\mathrm{MD}}=0.065{P}_{\mathrm{orb}}^{5/4}\left[{M}_{\odot }\right];\,\,{R}_{\mathrm{MD}}=0.094{P}_{\mathrm{orb}}^{13/12}\left[{R}_{\odot }\right],$$

where *P*_orb_ is the orbital period in hours within the range 1.3 ≤ *P*_orb _≤ 9 (see equation 2.100 and its derivation in ‘Cataclysmic Variable Stars’ in ref. ^9^). For the orbital period of ASKAP J1745-5051, these correspond to

2 $${M}_{\mathrm{MD}}=0.096\pm 0.008\left[{M}_{\odot }\right];\,\,{R}_{\mathrm{MD}}=0.13\pm 0.01\left[{R}_{\odot }\right].$$

We use ‘A Modern Mean Dwarf Stellar Color and Effective Temperature Sequence’ (http://www.pas.rochester.edu/~emamajek/EEM_dwarf_UBVIJHK_colors_Teff.txt)^58^ to approximate the corresponding M6–M6.5 spectral type and ~2,800 K temperature.

The second approach relies on the expected mass range from the spectral types corresponding to the fitted temperatures. We fit a blackbody function to the available photometric data from our observations and archival sources (see the Supplementary Information and Supplementary Table 1 separately for wavelengths below and above *λ* = 500 nm; Extended Data Fig. 5). Archival measurements were obtained from VizieR^70^ for GALEX, Gaia and the SkyMapper Southern Survey^71^.

We found that the short-wavelength data correspond to a blackbody temperature of 26,641 ± 4,139 K, with a reduced *χ*^2^ of 4.27 (Supplementary Information). This temperature is unusually high for a white dwarf in a CV below the period gap, where the mean temperature is of order 15,000 K (ref. ^72^). For a similar outlier CV SDSS J153817.35+512338.0, with an effective temperature of $35\,28{4}_{-688}^{+600}\,{\rm{K}}$ and a period of ~1.5 h, it is thought that the high temperature may be the result of a recent nova outburst or ongoing accretion onto the white dwarf^72,73^. ASKAP J1745-5051 exhibits features of ongoing accretion and hints, in the asynchronous polar scenario, of a possible post-nova outburst disequilibrium. The temperature of ASKAP J1745-5051 may therefore indicate a heightened period of accretion onto the white dwarf and provide evidence for a possible evolutionary link between LPTs and AR Sco-like systems^15,16^. From the long wavelength data, we obtained a temperature of 2,781 ± 59 K with a reduced *χ*^2^ of 130.53. This fitted temperature is consistent with the effective temperature, and therefore companion spectral type, estimated from the mass–period relationship. We note that the blackbody fitting may be impacted by contamination from an accretion disk/stream, the nearby star Gaia 1488 or possibly both. This contamination, along with the poorly constrained blackbody fit, may imply higher than reasonable brightness at longer wavelengths.

With these mass estimates, we calculate the orbital centre of mass separation from Kepler’s third law^9^

3 $$a=3.53\times 1{0}^{10}{M}_{{\rm{WD}}}^{1/3}{(1+{M}_{{\rm{MD}}}/{M}_{{\rm{WD}}})}^{1/3}{P}_{{\rm{orb}}}^{2/3},$$

where the masses are normalized to solar mass units, *P*_orb_ is in hours and the separation *a* is in cm. Using the orbital period *P*_orb_ = 1.369, the median white dwarf mass *M*_WD_ = 0.83 *M*_⊙_, and the companion mass calculated above, we obtained

$$a=4.24\pm 0.37\times 1{0}^{10}\,\mathrm{cm}=0.61\pm 0.05\,{R}_{\odot }.$$

We similarly used the mass estimates and orbital period to calculate the binary mass function

4 $$f=\frac{{M}_{\mathrm{WD}}^{3}{\mathrm{sin}}^{3}i}{{({M}_{\mathrm{WD}}+{M}_{\mathrm{MD}})}^{2}}=\frac{{P}_{\mathrm{orb}}{K}^{3}}{2{{\pi }}G},$$

where *i* is the inclination of the orbit and *G* is the gravitational constant. For this calculation, we also used the radial velocity semi-amplitude *K* = 114.2 ± 7.5 km s^−1^ obtained from The Joker. In Extended Data Fig. 6, we show how this constrains the inclination of the system to be near face-on.

### Dynamic spectra

To explore the time and frequency structure of ASKAP J1745-5051 radio emission, we formed dynamic spectra from the observations with ASKAP, ATCA and MeerKAT using DStools (https://github.com/askap-vast/dstools/, ref. ^74^). For each observation, we first used WSCLEAN^75^ and CASA^76^ to image and self-calibrate the data and produce a model of all sources of emission within the primary beam, masking the position of ASKAP J1745-5051 from the model. We then formed model-subtracted visibilities and baseline-averaged the data to produce dynamic spectra, discarding visibilities on baselines shorter than 500 m to reduce the impact of poorly modelled diffuse emission and radio frequency interference.

We used the RM-LITE (https://github.com/AlecThomson/rm-lite/) implementation of RM-Tools^77^ to improve the signal-to-noise ratio of linearly polarized emission via rotation measure (RM) synthesis^78^ and to correct for Ricean bias^79^ of the linearly polarized intensity. Finally, we rebinned the data to an optimal time and frequency resolution for each observation, and generated frequency-averaged visibility lightcurves in all Stokes parameters.

We did not detect quiescent or bursting emission in the single 6-h ATCA C/X-band observation above a 3*σ* limit of 268 μJy per beam (ATCA Epoch 9). In L-band observation ATCA Epoch 2, we detected a quiescent source showing no significant variability or bursting emission on shorter timescales. In all other observations (ASKAP, ATCA Epoch 1, ATCA Epochs 3–8 and MKT Epochs 1–3), we detected multiple highly elliptically polarized bursts, showing varying degrees of fractional polarization, intermittency and burst substructure. The properties of each burst are summarized in Supplementary Table 1.

In Fig. 3, we show Stokes *I* dynamic spectra from the ATCA Epoch 4 and ATCA Epoch 8 observations, along with frequency-averaged lightcurves in Stokes *I*, Stokes *V* and linearly polarized intensity $\mathrm{PI}=\sqrt{{Q}^{2}+{U}^{2}}$. In the ATCA Epoch 4 observation, we detected three sets of bursts appearing as double-peaked pulses. The burst sets are separated by ~1.3 h while the separation between each component narrows from ~30 min to ~10 min. The pulses have a lower frequency cut-off near 1,500–1,700 MHz, and upper cut-off around 2,500–2,600 MHz, and drift upwards in frequency with time at a rate of ~1–2 MHz min^−1^. We do not detect any pulsed emission from ASKAP J1745-5051 for the remaining ~7 h following the three detected burst sets.

In ATCA Epoch 8, we detected bursts occurring throughout the 15-h observation. While less intermittent than in ATCA Epoch 4, the pulses in this observation show a similar modulation of lower and upper frequency cut-off and strong pulse-to-pulse variability. The upper frequency cut-off appears to drift between 2,500 MHz and 2,700 MHz sinusoidally over a period of ~8 h.

In Fig. 4, we show the Stokes *I* dynamic spectrum of four pulses detected in the 1,300–1,500 MHz subband of the MKT Epoch 1 observation. The pulses exhibit multipeaked profiles that vary significantly in both total intensity and polarization over the observation. The pulses have narrow-band frequency structure, with peaks spaced apart by ~15–35 MHz, decreasing in spacing towards the top of the band. The peaks increase in width from approximately 1 to 10 MHz, and drift downwards in frequency at a rate of 1 MHz min^−1^ within each pulse. In some cases, the drift continues between pulses with peaks appearing to smoothly connect from one pulse to the next.

### Pulse periodicity

We used the dynamic spectra extracted with DStools from ATCA, MeerKAT and ASKAP observations to produce Stokes *I* lightcurves. For each observation with multiple pulses detected, we extracted the pulse periodicity using a Lomb–Scargle periodogram. We used the nifty-ls^80^ implementation of the astropy Lomb–Scargle method^81^. For each frequency peak in the periodogram, we take the half width half maximum as the nominal frequency uncertainty.

In this analysis, we found a radio period of *P*_radio_ = 1.345 ± 0.084 h combining the lightcurves from ATCA Epochs 6–8 (Supplementary Fig. 2). For the combined MKT Epochs 1–3, we determined an initial radio period of *P*_radio_ = 1.31 ± 0.13 h. Similarly for the Einstein Probe data, binned to 200 s resolution, we find an X-ray period of *P*_X_ = 1.32 ± 0.13 h. We used the same Lomb–Scargle approach with the scipy.signal^82^ implementation of a Savitzky–Golay filter, using a first-degree polynomial and a filter size of 30 to smooth the noisy power spectrum (Supplementary Fig. 3).

Using the initial period to fold the radio lightcurves, we then measured pulse times of arrival (ToAs) to determine a more accurate period using pulsar timing techniques (see the Supplementary Information for further details). Fitting the ToAs across the ATCA and ASKAP detections with PINT^83^, we determined a period of ${P}_{\mathrm{radio}}=1.3449{7}_{-0.00004}^{+0.00003}\,{\rm{h}}$ ($4,841.{89}_{-0.14}^{+0.12}\,{\rm{s}}$). The ToAs have a phase-connected solution across an ~2-year baseline. We also obtained an upper limit on the period derivative of $\mathop{P}\limits^{^\circ }$ < 1.5 × 10^−8^ s s^−1^, taking the 95% absolute value.

We found that the radio periods, as well as the Einstein Probe X-ray period, are all consistent with both the ToA period, and the spectroscopic orbital period for ASKAP J1745-5051. Furthermore, we found that the X-ray emission appears to be phase-aligned with the MeerKAT pulses, but anti-phase with the ATCA and ASKAP pulses (see the Supplementary Information for phase delay calculations).

### Pulse polarization

All ASKAP J1745-5051 pulses feature a high degree of elliptical polarization, with a linear fraction of 23–97% and an absolute circular fraction of 0–56%. None of our detected pulses show a significant RM. The polarization PA is typically 20–40° and remains flat or slowly wanders over the pulse profile, although on short timescales the polarization state often undergoes significant variability.

In Extended Data Fig. 2, we show the polarization characteristics of a multicomponent pulse detected in the MKT Epoch 1 observation, beginning at 2024-12-27 03:00 UTC. In the initial stages of this pulse, the polarization state is ~80% linear and ~0% circular, with a PA of 35°. This state is retained during a sharp drop in total intensity at 13 min, and emission then transitions to 50% linear and 50% circular as the next pulse component builds in intensity. From 22 to 24 min, the total intensity dips and the emission becomes significantly depolarized. As the emission returns to a highly polarized state, the PA swings through two complete revolutions of the Poincaré sphere with a constant fractional circular polarization. The emission then returns to the initial polarization state at 26 min and stays constant as the pulse intensity decays. Further analysis of the polarization properties from ASKAP J1745-5051 will be published in a follow-up paper.

### Pulse substructure

We conducted a single-pulse search of the 2-h Murriyang data using PRESTO (https://github.com/scottransom/presto). A dispersion measure (DM) range of up to 350 pc cm^−3^ was used—double the maximum Galactic DM for a distance of 6 kpc from the NE2001 electron density model^84^. Searching in the 1,100–2,600 MHz frequency range, we found no convincing pulsar-like candidates. We provide further details on a periodicity search in the Supplementary Information.

We used MeerKAT’s PTUSE backend to record and search the full Stokes data for subburst structure on timescales of 300 μs to 300 ms. We conducted a single-pulse search of the PTUSE data from MKT Epochs 1–3 using the HEIMDALL package (https://sourceforge.net/projects/heimdall-astro/, ref. ^85^). The search covered a DM range of 0–1,000 pc cm^−3^ with a DM tolerance factor of 1.1, and a detection threshold of signal-to-noise ratio ≥7. We did not find any evidence of subburst structure on timescales of 100 μs to 7 s. See the Supplementary Information for details on candidate inspection.

## Modelling

### Emission mechanism

Assuming a conservatively large *r* = 1 *R*_⊙_ upper limit on the emission region, a minimum distance of 0.4 kpc and a typical flux density of *F*_*ν*_ = 1 mJy at 1 GHz, pulses from ASKAP J1745-5051 have a brightness temperature of at least

5 $${T}_{{\rm{B}}}=\frac{{F}_{\nu }{c}^{2}}{2{k}_{{\rm{B}}}{\nu }^{2}\varOmega } > 1{0}^{12}\,{\rm{K}},$$

where Ω = πarctan^2^(*r*/*d*) and *ν* is the central observing frequency. This lower limit necessitates a coherent emission mechanism. We note that for all Gaia parallax and Bailer–Jones photogeometric distances, *T*_B_ exceeds the 10^12^ K Compton limit. The observed pulsed emission reaches high degrees of fractional polarization nearing 100% and features a slowly varying PA. This implies that the emission must originate from a region with highly ordered magnetic fields; otherwise, the superposition of emission from randomly oriented field structures would lead to substantial depolarization (for example, ref. ^86^). This further constrains the emission region of ASKAP J1745-5051 pulses to a much smaller region than 1 *R*_⊙_ and a correspondingly larger lower limit on the minimum *T*_B_. Therefore, we do not consider incoherent mechanisms such as gyrosynchrotron emission as they cannot produce the observed brightness temperatures in excess of 10^12^ K. Nevertheless, we note that some radio emission from AR Sco-like systems and CVs may be produced by gyrosynchrotron processes (for example, ref. ^87^).

We instead propose ECME generated from a relativistic electron population (for example, ref. ^45^) as a likely candidate to explain the observed radio properties of ASKAP J1745-5051. ECME can produce high brightness temperatures with large circular or elliptical polarization when electrons develop a population inversion in the velocity distribution in the presence of a strong magnetic field (for example, ref. ^88^). High degrees of linear polarization, as observed here, can arise when the emitting electrons have relativistic energies, shifting the emission from purely circular to elliptical polarization.

Our optical spectra and X-ray observations show evidence for ongoing accretion, and the presence of strong HeII emission lines is suggestive of accretion via magnetically channelled streams. These accretion streams can provide the necessary ingredients for the production of relativistic ECME: a source of energetic electrons, a means to develop a population inversion in the converging magnetic field of the accretion stream, and a mechanism to accelerate electrons to relativistic energies. We note that the electron density lower limit from the accretion region is three orders of magnitude higher than the *n*_e_ < 9 × 10^10^ cm^−3^ upper limit, inferred from the plasma frequency condition (Supplementary Information). This provides additional evidence that the ECME is produced in a lower density plasma region, not cospatial with the origin of the Balmer line emission.

### Simulated dynamic spectra

We modelled the observed radio pulse behaviour using a geometric simulation of interacting magnetic dipoles representing a magnetic white dwarf and M dwarf in an asynchronous binary system—implemented with PYVISTA^89^ (see the Supplementary Information for simulation details). By tracing the combined magnetic field topology and identifying regions satisfying the conditions for ECME, we generated synthetic dynamic spectra that reproduce multiple key features of the observations (Extended Data Fig. 7). The model produces double-peaked radio pulses recurring at the same orbital phases immediately before and after inferior conjunction of the white dwarf, variation in the spacing of each pulse pair, modulation of the upper and lower frequency cut-offs, and intermittency persisting over a timescale spanning multiple orbits. The intermittent phases occur when the white dwarf magnetic pole rotates away from the M dwarf, causing the flux tube connection to become disrupted.

These results show that several of the observed radio emission features from ASKAP J1745-5051 can arise purely from the evolving geometry of the interacting magnetic fields, although additional unmodelled effects due to gravitational influence, plasma flows and variable particle acceleration are expected to further shape the emission. The same changes in magnetic connectivity that determine radio pulse visibility may also modulate the accretion rate, providing an explanation for the observed differences in X-ray brightness between our Einstein Probe and Swift observations. Continued coordinated monitoring in both bands will test whether the radio and X-ray variability share this common origin.

## Supplementary information

Supplementary Information Supplementary Figs. 1–3, Tables 1–4 and text.

Peer Review File

Supplementary Data 1 This data file contains the arrival time, duration, fluxes and polarization properties for the fitted peaks across all observations summarized in Extended Data Table 1.

## Figures and Tables

**Extended Data Table 1:**
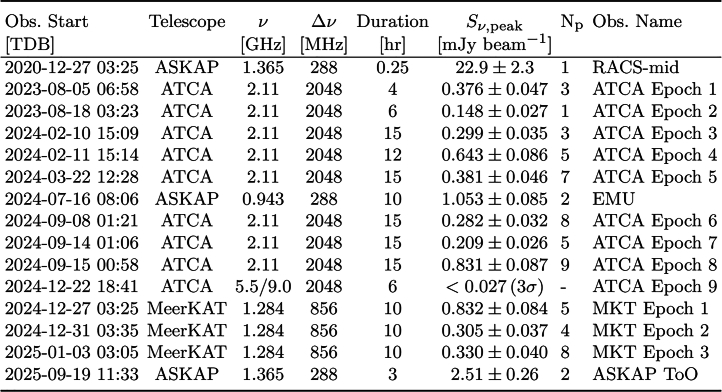
Radio detections of ASKAP J174508.9-505149

We include the barycentric observation start time, duration, central frequency *ν*, and bandwidth *Δν*. S*ν*,peak is the fitted peak time- and frequency-averaged flux density from the total intensity continuum image, and Np is the number of pulses detected during the observation above 3*σ*. Flux density errors are the quadrature addition of the fitted error, RMS, and brightness uncertainty scaling - 6% for ASKAP and 10% for ATCA and MeerKAT. We also include shorthand names used throughout the text for reference. Please see Supplementary Data 1 for a machine-readable version of this table.

## Data Availability

The ASKAP data used in this Article are available via the CSIRO ASKAP Science Data Archive (CASDA) at https://data.csiro.au/collections/domain/casdaObservation/search/ (ref. ^90^) under project codes AS110, AS107 and AS113. The ATCA data used in this Article are available via the Australia Telescope Online Archive (ATOA, https://atoa.atnf.csiro.au/query.jsp) under project codes C3363, CX553 and C3587. The Murriyang Parkes data (Project ID: PX113) are also accessible via the ATOA website. This Article makes use of data from MeerKAT (project ID: SCI-20241101-KR-02), which can be obtained through the SARAO archive (https://archive.sarao.ac.za). Other auxiliary datasets can be made available upon request via email to the corresponding author. The data that support the findings of this study are available via Zenodo at 10.5281/zenodo.17365566 (ref. ^91^).

## Code availability

Code to reproduce all figures and main results is available via GitHub at https://github.com/krose098/askap_j1745. Additional Python scripts used in the data reduction and analysis are available on request from K.R.
