# Supplementary material for: Periodic radio and X-ray emission from an accreting white dwarf binary
Source: Nat Astron. 2026 Jun 1;10(8):1166–78. doi: 10.1038/s41550-026-02882-x (PMC13476169; doi:10.1038/s41550-026-02882-x)
Supplement: Supplementary file 1 [file 41550_2026_2882_MOESM1_ESM.pdf]

nature astronomy

Supplementary information https://doi.org/10.1038/s41550-026-02882-x

Periodic radio and X-ray emission from an accreting white dwarf binary

In the format provided by the authors and unedited

Supplementary Information

Gaia Astrometric Uncertainty

The astrometric solution for ASKAP J1745−5051 in *Gaia* DR3 [1] indicates significant unreliability in the derived parallax distance, proper motion, and space velocity. The measured parallax of $\varpi = 1.75 \pm 0.91$ mas corresponds to a signal-to-noise ratio of ~ 2, resulting in a simple distance inversion ($d = 1/\varpi$) that is unreliable [2, 3]. Furthermore, the *Gaia* astrometric quality indicators for give an `astrometric_excess_noise` value of 3.84 mas (typically of order 0.1–1.0 mas), indicating substantial excess scatter in the along-scan residuals that cannot be accounted for by the measurement uncertainties alone [4, 5]. This is consistent with a photocentre wobble induced by an unresolved binary companion, which we have demonstrated is the case for *Gaia* 4032. Similarly, the `astrometric_sigma5d_max` value of 1.43 mas exceeds the recommended threshold of ∼1 mas for good astrometric solutions [5]. Together, these indicators imply that the reported proper motion measurements ($\mu_\alpha = 2.98 \pm 0.85\,\mathrm{mas\,yr^{-1}}$, $\mu_\delta = 3.77 \pm 0.75\,\mathrm{mas\,yr^{-1}}$) have underestimated uncertainties, and any derived tangential velocity is unreliable.

For these reasons we do not adopt the naive parallax inversion distance, and instead adopt a conservative range for ASKAP J1745−5051. As a lower bound we adopt $d_{\mathrm{min}} = 1/(\varpi + \sigma_\varpi)$ pc, corresponding to the 1σ parallax limit, and as an upper bound we adopt the 84th percentile of the "Bailer-Jones" photogeometric distance posterior [6]. This method uses a direction-dependent prior on the Milky Way stellar distance distribution and incorporates *Gaia* photometry alongside the parallax. As noted in the Methods, it is considered more reliable for *Gaia* sources with fractional parallax uncertainties in the range $0.1 \leq \sigma_\varpi/\varpi \leq 1$ [6], as is the case for *Gaia* 4032.

Therefore, combining the parallax and photogeometric distances, we adopt the distance range 0.4–9.1 kpc. This range is intended to conservatively constrain the true distance of ASKAP J1745−5051 given the unreliable astrometric solution and the binary nature of the system.

MALS

Each MALS observation has a 56 min integration time from three combined 1120 s scans. The MALS catalogue provides 15 100″ cutout images, each covering a 60 MHz spectral window within the band. We find eight nominal detections in 15 of the sub-band cutouts between 900–1400 MHz, with non-detections between 1250–1350 MHz and above ∼1500 MHz. Six of these detections, are above the 3σ threshold, with a median root mean square (RMS) of background noise σ =0.21 mJy beam^{-1}. We fit these detections, with fluxes ranging from 0.62–1.82 mJy beam^{-1}, to a simple $F(\nu) \sim \nu^\alpha$ power-law and obtain a spectral index of $\alpha = 2.68 \pm 0.29$. Further analysis of the archival MALS data was not undertaken as it would not have added to this work.

LDSS-3 Full Reduction

Wavelength calibration was performed using He, Ne, and Ar arc lamp exposures. Initial line identification was carried out on the central spectrum, followed by a correction for spectral curvature across the spatial direction. A two-dimensional master flat was generated in each night from dome flat exposures. We then modelled the lamp spectrum and divided that out to isolate the pixel-to-pixel sensitivity variations and the non-uniformity in the slit. Cosmic rays are identified and removed using the L.A.Cosmic algorithm [7]. We also acquire standard star observations, specifically of LTT 3864, to perform flux calibration. For each science exposure, a sky model is constructed with an iterative two-dimensional b-spline function, with a break points spacing of 1.0 pixel in the wavelength direction, and a 5th order polynomial in the spatial direction. The resulting sky-subtracted frames, along with their inverse variance arrays, are rectified onto a uniform wavelength and spatial grid. Spectra are then linearly resampled onto a common grid spanning 3000–10 500 Å. Science targets are extracted using a 10-pixel wide aperture. The spatial scale is 0.189″ per pixel. For standard stars, a circular extraction aperture with a 2 pixel radius is used. Telluric features are modelled and removed using `MOLECFIT` [8, 9], assuming H_2O and O_2 as the dominant molecular contributors. The absorption bands used in the telluric correction span 6820–6970 Å, 7210–7330 Å, 7590–7690 Å, 8170–8360 Å, 8170–8360 Å, and 9100–9400 Å.

Additional Swift Details

The first *Swift* observation (ID00016563001) was allocated 1.5 ks but only 0.24 ks was observed (starting at 2024-03-12 23:07 UTC) due to *Swift* downtime.

We used the `uvotdetect` tool from `HEASoft` to identify sources in the UVOT images. We input the sky image and exposure map for each of the UVOT bands and set the standard detection threshold of 3σ, where σ is defined as a standard deviation of the noise. This produced a catalogue of sources and photometry for each of the three images.

We filtered the `uvotdetect`-generated source catalogues for sources within 5″ of ASKAP J1745−5051's position. In the first observation (2024-03-12 23:07 UTC) we did not detect any coincident $\geq 3\sigma$ sources; likely due to the short exposure time.

In Supplementary Table 2 we convert the *Swift* magnitudes to the AB system using the additive corrections factors $1.51, 1.69, 1.73$ for the UVOT filters UVW1, UVM2, and UVW2, respectively; following the ABzeropoint *Swift* UVOT calibration document.

For the PIMMS calculation we assumed a photon power-law index of 2 and Galactic Hydrogen column density of $\mathrm{n_H} = 1.4 \times 10^{21}\,\mathrm{cm}^{-2}$, using the standard scaling relation [10] with an estimated maximum extinction of $A_V = 0.6\,\mathrm{mag}$.

Additional Line Fitting

We used the sum of a linear function and a Lorentzian to fit each spectral peak within a ± 100 Å range of the vacuum rest wavelength $\lambda_{\rm rest}$ of the respective emission line, using the `astropy` [11] implementation of the Levenberg-Marquardt algorithm and least squares statistic `LevMarLSQFitter`.

We calculated the equivalent widths by summing the values of the fitted Lorentzian within ± 50 Å of $\lambda_{\rm rest}$ and normalising by the average of the linear continuum fit across that range.

Plasma Frequency

A condition for the production of ECME at a frequency $\nu_{\rm B}$ is that $\nu_{\rm B} > \nu_{\rm p}$, where $\nu_{\rm p}$ is the plasma frequency. Since $\nu_{\rm p} \approx 9 \times 10^3 {n_{\rm e}}^{1/2}$ [12], the 2.7 GHz upper frequency cutoff of the ECME implies an electron density upper limit of $n_{\rm e} < 9 \times 10^{10}\,{\rm cm}^{-3}$.

Another characteristic of ECME is that $\nu_{\rm B} \approx 2.8 \times 10^6 B$ [12], where B is the magnetic field strength. Using the same 2.7 GHz upper frequency cutoff gives us the lower limit of $B > 0.96$ kG in the base of the emission region, which is consistent with the magnetic field strength expected in the interaction region.

Blackbody Fitting

We use the blackbody function

$$B(\lambda) = \frac{2hc^2/\lambda^5}{\exp(hc/(\lambda k_B T) - 1)}, \tag{1}$$

where h is the Planck constant, c the speed of light, k_B the Boltzmann constant, and T the temperature and assess the fit with a reduced χ^2 statistic

$$\chi^2 = \frac{1}{N-2} \sum \left(\frac{F_{\rm obs} - F_{\rm fit}}{\Delta F_{\rm obs}} \right)^2, \tag{2}$$

where $F_{\rm obs}$ and $F_{\rm fit}$ is the observed and fitted flux, respectively, N is the number of measurements, and $\Delta F_{\rm obs}$ are the uncertainties on the observed flux.

We use the `curve_fit` method from the `scipy.optimize` library [13].

We apply an extinction correction of $A_V = 0.6$ mag to the short wavelength ($\lambda \leq 500$ nm) fluxes to obtain the AB magnitudes listed in Supplementary Table 1. The flux scaling was calculated with the extinction Python package

Pulse Sub-Structure Analysis

We carried out a periodicity search of the Murriyang data with `pulsar_miner` – an automated pulsar searching pipeline based on `PRESTO`. For computational efficiency we performed an initial test for periodic pulses followed by a more detailed search for single pulses. In the first case, we searched for pulses in the first 15 min. We used a dispersion measure (DM) range of up to

$300\,\mathrm{pc\,cm^{-3}}$ – double the plausible Galactic DMs along line-of-sight based on the YMW16 electron density model [14] – and periods ranging from 1 ms to 10 s. We performed acceleration searches in the Fourier domain using the `accelsearch` routine, with a maximum allowed Doppler-induced Fourier bin drift $z_{\max} = \pm 200$. We found no convincing pulsar-like candidates in our search with a threshold of 8.

From the PTUSE search, we obtained $\sim 3 \times 10^4$ initial candidates. These were subsequently reduced to $\sim 5 \times 10^3$ candidates using the machine-learning classifier `FETCH` [15], which evaluates candidates based on their time–frequency morphology. The candidates were further processed with the RFI removal tool clfd [16]. We then inspected the remaining events manually and verified with the `pdmp` tool from `PSRCHIVE` [17] that all of them correspond to zero-DM signals, consistent with terrestrial radio-frequency interference. We therefore conclude that no astrophysical short duration bursts are present in these data.

Time of Arrival Analysis

We used a triangle-shaped pulse model, with variable height, phase width, and centroid to measure times of arrival (ToAs) from the ATCA and ASKAP observations, adopting the centroid as the derived ToA. For observations exhibiting a double-peaked profile, we use a double-triangle model, and arbitrarily adopt the flux-weighted centroid as the ToA. We used time-integrated, folded pulse profiles per observation to measure the ToAs, which we produced using a weighted average for each observation, weighted by the single-pulse signal-to-noise ratio. This improved the ToA precision while mitigating against decreases in flux density observed during some observations.

We fit the timing model using PINT [18]. We first performed an initial fit to the ToAs with a simple timing model where only the spin frequency ν was fit as a free parameter. After establishing a stable solution for the spin period alone, we then allowed the spin frequency time derivative $\dot{\nu}$, along with so-called EFAC and EQUAD parameters, which we used to augment the ATCA ToA uncertainties, to vary. EFAC and EQUAD are parameters used in pulsar timing analyses [e.g., 19] that adjust the ToA uncertainties (σ_{ToA}) multiplicatively and in quadrature as $\sigma_{\mathrm{ToA}} \rightarrow \mathrm{EFAC}\sqrt{\sigma_{\mathrm{ToA}}^2 + \mathrm{EQUAD}^2}$.

We find $\nu = 0.000206529^{+6\times10^{-9}}_{-5\times10^{-9}}$, $\dot{\nu} < 5.7 \times 10^{-16}$ at 95% confidence, $\mathrm{EFAC} = 0.8^{+0.7}_{-0.2}$, and $\log_{10}(\mathrm{EQUAD/s}) = -2^{+3}_{-2}$, with a period epoch of MJD 60350.9997.

Simulated Dynamic Spectra Details

Synthetic dynamic spectra were generated with a geometric model implemented in PyVista [20], representing the system as two magnetic dipoles of moments $7 \times 10^{32}\,\mathrm{G cm^3}$ (white dwarf) and $2 \times 10^{33}\,\mathrm{G cm^3}$ (M dwarf) separated by $0.61\,\mathrm{R_\odot}$. These correspond to surface polar field strengths of 4 MG and 1.2 kG for stellar radii of $0.01\,\mathrm{R_\odot}$ (white dwarf) and $0.2\,\mathrm{R_\odot}$ (M dwarf),

respectively. The binary was evolved in 1 min timesteps over a circular orbit with period 1.35 h, adopting a synchronised M dwarf rotation period and an asynchronous white dwarf rotation period of 1.33 h. The spin axes of both stars and the M dwarf magnetic axis were aligned with the orbital axis, while the white dwarf magnetic axis was inclined by 10°. The system inclination was set to 15°.

We computed the three-dimensional magnetic vector field at each timestep and traced field lines along a flux tube initially connecting the M dwarf's northern magnetic hemisphere to the white dwarf's southern magnetic pole. Voxels within the flux tube were tested for viable ECME generation and visibility, requiring the local magnetic field vector to lie within ±5° of perpendicular to the observer's line of sight and to fall within a prescribed acceleration region extending from roughly 70–75 % along the tube length toward the white dwarf. This region was chosen to be consistent with the presence of relativistic electrons implied by the observed elliptical polarisation of the radio pulses. The resulting synthetic dynamic spectrum assigns each frequency–time bin a value of 1 when at least one visible emission site exists with a local field strength matching that ECME frequency, and 0 otherwise — indicating where ECME generation and visibility are viable rather than the emitted intensity.

Several model parameters – such as the magnetic field strengths, white dwarf rotation period and resulting beat cycle, the inclination of the white dwarf magnetic axis, orbital inclination, and emission beaming angle – cannot be uniquely constrained in these simulations. We therefore adopted values consistent with the observed orbital geometry and within plausible physical ranges for magnetic cataclysmic variables. This model explores only the geometric aspects of the emission process; additional effects due to gravitational influence, plasma flows and supply, and variations in the electron distribution are expected to further modify the observed emission properties, and may explain departures from our simulated dynamic spectra.

Orbital Phase Delay

To obtain the X-ray orbital phase we fit a sinusoid to the phase-folded *Einstein Probe*-FXT data, with all three observations binned to 50 s time resolution; shown in Fig. 2.We obtain the peak orbital phase $\phi_X = 0.89 \pm 0.19$ at the maximum of the fitted curve, and take the uncertainty to be the square root of the phase term from the `curve_fit` covariance matrix [13].
To calculate the orbital phase delays we take the difference in phases and use the quadrature sum of their respective uncertainties as the delay uncertainty:

$$\text{X-ray vs. MKT: } \Delta\phi = 0.89 - 0.8 \pm \sqrt{0.19^2 + 0.1^2} = 0.09 \pm 0.21$$
$$\text{X-ray vs. ATCA/ASKAP: } \Delta\phi = 0.89 - 0.31 \pm \sqrt{0.19^2 + 0.03^2} = 0.58 \pm 0.19$$
$$\text{X-ray vs. RV: } \Delta\phi = 0.89 - 0.50 \pm \sqrt{0.19^2 + 0.32^2} = 0.39 \pm 0.37$$

We see that the phase delay between the MeerKAT pulses and the X-ray peak is negligible. Similarly the phase delay between the X-ray and radial

velocity posterior is not significant. However the ATCA and ASKAP pulses are anti-phase with respect to the X-ray emission, with a nearly 3σ significance.

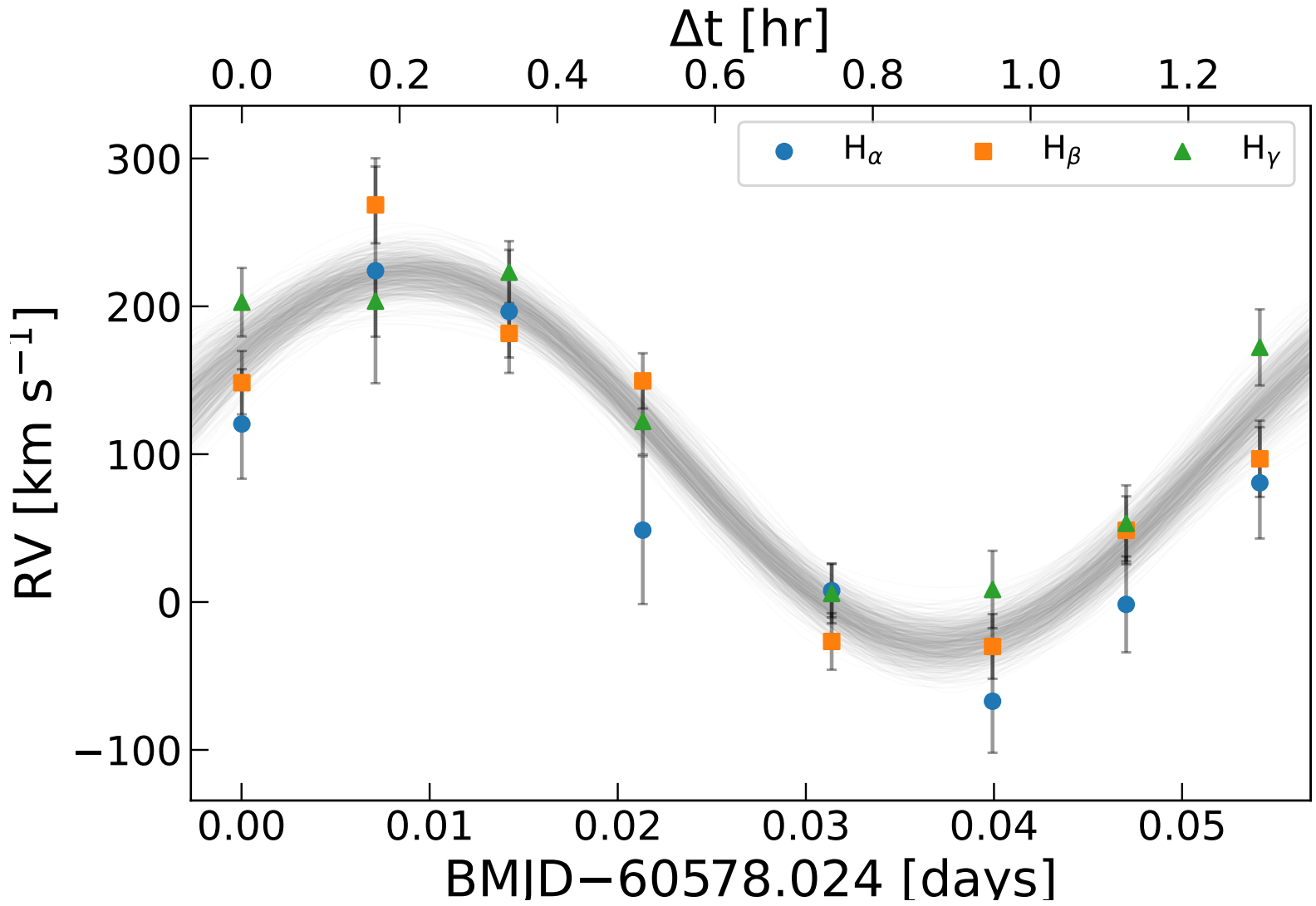

Supplementary Figure 1: Radial velocity measurements from *SOAR* observations with 1σ error bars around the fitted values. We show posterior sample curves from `TheJoker` in grey. We use circle, square, and triangle markers for the H_α, H_β, and H_γ radial velocity measurements, respectively.

205 # Supplementary Tables

Supplementary Table 1: AB magnitude photometry for ASKAP J1745−5051 from archival UV and optical as well as UV bands from the *Swift* observation (2024-05-16 17:43 UTC). We note the central wavelength λ_{cent} the instrumental filter name.

λ_{cent} [nm]	AB Magnitude [mag]	Instrument	Filter
152.8	21.36 ± 0.149	GALEX	FUV
192.8	21.792 ± 0.163	Swift	UVW2
224.6	21.153 ± 0.154	Swift	UVM2
231.0	21.36 ± 0.105	GALEX	NUV
260.0	21.732 ± 0.201	Swift	UVW1
518.26	18.973 ± 0.102	Gaia	G_BP
617.0	18.511 ± 0.042	SkyMapper	r_psf
639.07	19.64 ± 0.039	Gaia	G
782.51	18.311 ± 0.077	Gaia	G_RP
916	17.895 ± 0.047	SkyMapper	z_psf

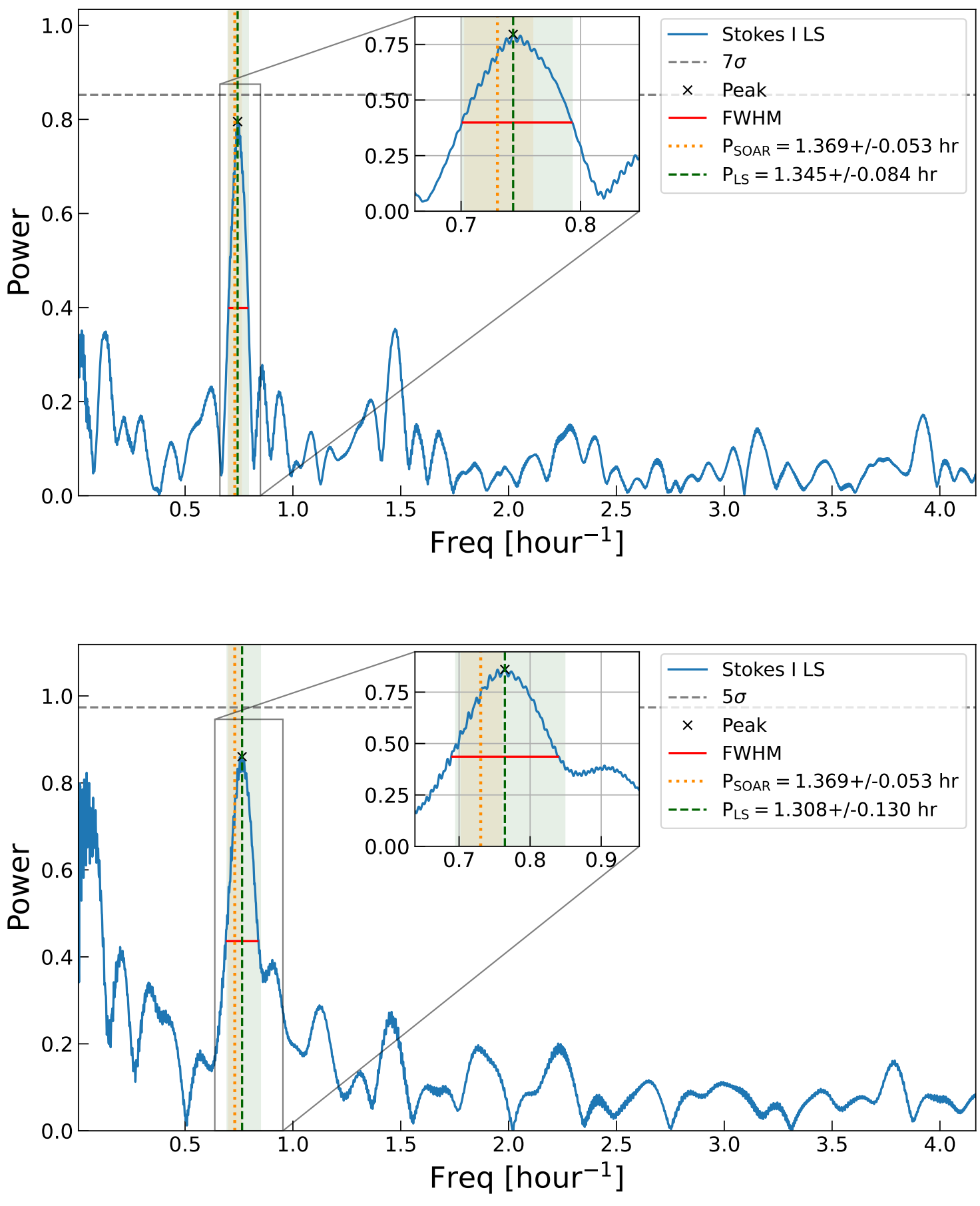

Supplementary Figure 2: Lomb-Scargle Periodograms of ASKAP J1745−5051 radio observations. We show combined ATCA Epochs 6–8 (Top) and MKT Epochs 1–3 (Bottom). The dashed green line shows the Lomb-Scargle period and the dotted orange line shows the period extracted from the *SOAR* radial velocity fitting.

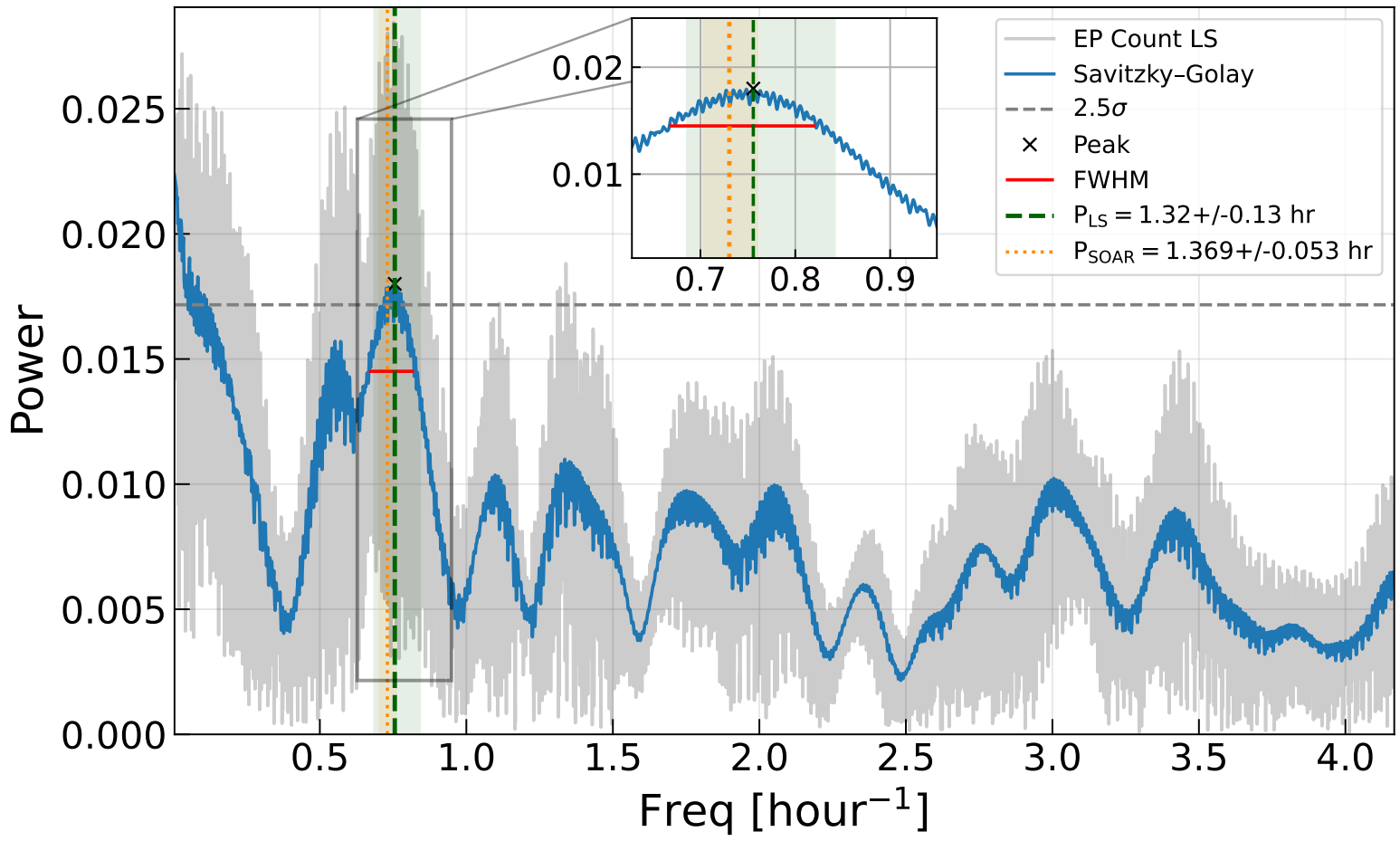

Supplementary Figure 3: Smoothed Lomb-Scargle Periodogram of combined *Einstein Probe* observations of ASKAP J1745−5051 binned with 200 s time resolution. The dashed green line shows the Lomb-Scargle period and the dotted orange line shows the period extracted from the *SOAR* radial velocity fitting.

Supplementary Table 2: Photometric rates, magnitudes, and fluxes for the different *Swift* UVOT bands in the second observation (2024-05-16 17:43 UTC). We also calculate the angular separation between the positions of ASKAP J1745−5051 in RACS-mid and each of the sources identified with `uvotdetect`.

Filter	Rate [count/s]	Vega Magnitude [mag]	Flux [erg s^{-1} cm^{-2}]	Sep. [$''$]
UVW1	0.299 ± 0.045	18.46 ± 0.16	$4.3 \pm 0.6 \times 10^{-13}$	0.79
UVM2	0.229 ± 0.033	18.17 ± 0.15	$5.6 \pm 0.8 \times 10^{-13}$	0.54
UVW2	0.168 ± 0.031	18.93 ± 0.20	$2.8 \pm 0.5 \times 10^{-13}$	0.32

Supplementary Table 3: Fitted Values for SOAR spectra

**Phase ϕ**	0.06	0.24	0.39	0.52	0.65	0.69	0.81	0.94
**Hα Line**								
RV [km/s]	120.4	224.1	196.5	48.6	7.7	-67.1	-1.6	80.6
err [km/s]	37.0	76.1	41.6	50.0	18.1	34.8	32.4	37.7
EQW [Å]	16.6	19.6	20.0	21.5	17.1	21.5	20.7	20.0
FWHM [Å]	21.7	40.4	29.0	32.9	15.6	23.7	21.0	22.5
**Hβ Line**								
RV [km/s]	148.3	268.6	181.7	149.5	-26.7	-30.0	48.5	96.9
err [km/s]	21.4	26.0	16.3	18.7	19.1	21.9	22.9	25.8
EQW [Å]	30.3	28.2	30.1	34.1	28.8	28.9	31.9	28.6
FWHM [Å]	16.6	19.9	16.2	18.5	18.1	16.9	18.9	18.8
**Hγ Line**								
RV [km/s]	202.8	203.5	223.1	122.2	5.8	8.5	53.2	172.2
err [km/s]	23.2	24.1	21.0	22.3	20.2	26.2	25.7	25.8
EQW [Å]	33.3	31.6	36.5	33.6	28.7	30.0	27.4	32.6
FWHM [Å]	18.1	19.0	20.1	20.1	17.5	18.1	17.3	18.7
**He II Line**								
RV [km/s]	134.5	179.2	169.3	22.4	-143.0	-74.0	37.5	212.1
err [km/s]	58.9	32.7	33.6	43.9	33.0	41.8	29.8	48.1
EQW [Å]	13.6	11.7	12.1	14.4	12.1	12.2	10.3	13.4
FWHM [Å]	17.2	11.9	12.8	16.7	13.4	13.2	9.9	15.5
**Line Ratios**								
Hα/Hβ	0.549	0.696	0.664	0.629	0.593	0.744	0.648	0.698
HeII/Hβ	0.450	0.415	0.402	0.423	0.418	0.420	0.324	0.468

Supplementary Table 4: Fitted Values for LDSS-3 spectra

**Phase ϕ**	0.34	0.38	0.47	0.52
**Hα Line**				
RV [km/s]	-81.2	-54.1	-251.8	-329.9
err [km/s]	240.9	232.8	154.6	156.9
EQW [Å]	21.1	17.3	27.9	28.5
FWHM [Å]	19.2	16.5	19.9	19.1
**Hβ Line**				
RV [km/s]	-148.0	-32.1	-188.3	-125.5
err [km/s]	38.3	42.8	23.5	31.4
EQW [Å]	28.7	26.7	47.6	31.9
FWHM [Å]	14.2	14.7	16.5	15.2
**Hγ Line**				
RV [km/s]	-136.3	-145.7	-129.7	-187.3
err [km/s]	14.8	13.0	12.6	19.1
EQW [Å]	32.0	22.0	27.1	29.0
FWHM [Å]	15.8	12.1	14.4	19.6
**Hδ Line**				
RV [km/s]	-156.3	-0.9	-431.3	-230.7
err [km/s]	6.4	6.0	6.7	6.4
EQW [Å]	14.7	9.3	13.6	9.8
FWHM [Å]	10.7	7.8	11.0	8.4
**He II Line**				
RV [km/s]	-159.2	27.7	-178.9	-137.6
err [km/s]	53.0	43.8	50.8	41.0
EQW [Å]	16.3	13.4	13.8	8.4
FWHM [Å]	13.4	10.9	14.6	8.9
**Line Ratios**				
Hα/Hβ	0.737	0.646	0.587	0.894
HeII/Hβ	0.568	0.502	0.291	0.262

References

[1] Gaia Collaboration *et al.* Gaia Data Release 3. Summary of the content and survey properties. *Astron. Astrophys.* **674**, A1 (2023).

[2] Bailer-Jones, C. A. L. Estimating Distances from Parallaxes. *Publ. Astron. Soc. Pac.* **127**, 994 (2015).

[3] Luri, X. *et al.* Gaia Data Release 2. Using Gaia parallaxes. *Astron. Astrophys.* **616**, A9 (2018).

[4] Lindegren, L. *et al.* The astrometric core solution for the Gaia mission. Overview of models, algorithms, and software implementation. *Astron. Astrophys.* **538**, A78 (2012).

[5] Lindegren, L. *et al.* Gaia Early Data Release 3. The astrometric solution. *Astron. Astrophys.* **649**, A2 (2021).

[6] Bailer-Jones, C. A. L., Rybizki, J., Fouesneau, M., Demleitner, M. & Andrae, R. Estimating Distances from Parallaxes. V. Geometric and Photogeometric Distances to 1.47 Billion Stars in Gaia Early Data Release 3. *Astron. J.* **161**, 147 (2021).

[7] van Dokkum, P. G., Bloom, J. & Tewes, M. L.A.Cosmic: Laplacian Cosmic Ray Identification. Astrophysics Source Code Library, record ascl:1207.005 (2012).

[8] Smette, A. *et al.* Molecfit: A general tool for telluric absorption correction. I. Method and application to ESO instruments. *Astron. Astrophys.* **576**, A77 (2015).

[9] Kausch, W. *et al.* Molecfit: A general tool for telluric absorption correction. II. Quantitative evaluation on ESO-VLT/X-Shooterspectra. *Astron. Astrophys.* **576**, A78 (2015).

[10] Güver, T. & Özel, F. The relation between optical extinction and hydrogen column density in the Galaxy. *Mon. Not. R. Astron. Soc.* **400**, 2050–2053 (2009).

[11] Astropy Collaboration *et al.* The Astropy Project: Sustaining and Growing a Community-oriented Open-source Project and the Latest Major Release (v5.0) of the Core Package. *Astrophys. J.* **935**, 167 (2022).

[12] Dulk, G. A. Radio emission from the sun and stars. *Annu. Rev. Astron. Astrophys.* **23**, 169–224 (1985).

[13] Virtanen, P. *et al.* SciPy 1.0: Fundamental Algorithms for Scientific Computing in Python. *Nature Methods* **17**, 261–272 (2020).

[14] Yao, J. M., Manchester, R. N. & Wang, N. A New Electron-density Model for Estimation of Pulsar and FRB Distances. *Astrophys. J.* **835**, 29 (2017).

[15] Agarwal, D., Aggarwal, K., Burke-Spolaor, S., Lorimer, D. R. & Garver-Daniels, N. Fetch: A deep-learning based classifier for fast transient classification. *Mon. Not. R. Astron. Soc.* **497**, 1661–1674 (2020). URL https://doi.org/10.1093/mnras/staa1856.

[16] Morello, V. *et al.* The high time resolution universe survey – xiv. discovery of 23 pulsars through gpu-accelerated reprocessing. *Mon. Not. R. Astron. Soc.* **483**, 3673–3685 (2018). URL https://doi.org/10.1093/mnras/sty3328.

[17] van Straten, W., Demorest, P. & Oslowski, S. Pulsar Data Analysis with PSRCHIVE. *Astronomical Research and Technology* **9**, 237–256 (2012).

[18] Luo, J. *et al.* PINT: A Modern Software Package for Pulsar Timing. *Astrophys. J.* **911**, 45 (2021).

[19] Lentati, L. *et al.* TEMPONEST: a Bayesian approach to pulsar timing analysis. *Mon. Not. R. Astron. Soc.* **437**, 3004–3023 (2014).

[20] Sullivan, B. & Kaszynski, A. PyVista: 3D plotting and mesh analysis through a streamlined interface for the Visualization Toolkit (VTK). *Journal of Open Source Software* **4**, 1450 (2019). URL https://doi.org/10.21105/joss.01450.
