# Supplementary material for: Periodic radio and X-ray emission from an accreting white dwarf binary
Source: Nat Astron. 2026 Jun 1;10(8):1166–78. doi: 10.1038/s41550-026-02882-x (PMC13476169; doi:10.1038/s41550-026-02882-x)
Supplement: Supplementary file 2 [file 41550_2026_2882_MOESM2_ESM.pdf]

nature portfolio

Peer Review File

Periodic Radio and X-ray Emission from an Accreting White Dwarf Binary

Corresponding Author: Mr Kovi Rose

This manuscript has been previously reviewed at another journal. This document only contains information relating to versions considered at Nature Astronomy.

This file contains all reviewer reports in order by version, followed by all author rebuttals in order by version.

Version 0:

Reviewer comments:

Reviewer #1

(Comments for the Author)
Summary

This manuscript presents interesting radio observations of a magnetic cataclysmic variable and should be published, although not in its current form and without major modifications. In this reviewer's opinion, the manuscript contains many inaccuracies, misrepresentations, and incorrect analyses, which should be addressed as specifically noted in the major and minor issues sections below. Note that due to the number of such issues, it is likely that the reviewer has not identified all of them.

In summary, the manuscript asserts that J1745 is a long period radio transient (LPRT), but does not define their characteristics to show that it is. It also asserts that the radio source is at a distance of 5.6 kpc, when the Gaia distance is an order of magnitude less at 570 pc. The nearer distance gives a more reasonable explanation for all of the observations, such as its space velocity and radio, optical, ultraviolet, and X-ray flux. The manuscript then asserts that electron cyclotron maser emission is the cause of the radio emission because of the high brightness temperature, when gyrosynchrotron emission of low harmonic number provides a more natural explanation of the coherent elliptically polarized emission. Finally, the manuscript asserts that J1745 is similar to the magnetic white dwarfs AR Sco, J1912, and J2306, which are non-accreting magnetic cataclysmic variables, whereas J1745 shows none of their observed radio or optical characteristics and does not have a rapidly rotating WD. The manuscript does conclude that J1745 is a magnetic cataclysmic variable with an asynchronously rotating WD, but does not explicitly state that it is likely an asynchronous polar.

Major Issues

In 111: Asynchronous polars usually have spin periods within a few percent of the orbital period and synchronize within a few decades. However, a few of them have larger asychronisms of ~15%. They do not have asynchronisms >20%. They would then be considered IPs. See e.g., Littlefield et al. 2023, ApJ 165, 43 and Littlefield et al. 2023, 943, L24. This discussion should be corrected.

In 114: AR Sco is not a typical IP. Typical IPs have spin periods of ~10% of the orbital period. See Warner 1995. AR Sco belongs to a subclass of rapidly-rotating magnetic cataclysmic variables (MCVs) that are non-accreting. Its strong synchrotron-powered radio emission is unusual for MCVs, unlike that of J1745. Most IPs show radio emission similar to polars, i.e., highly circularly polarized emission due to plasma emission or electron cyclotron maser emission (ECME). Therefore, this paragraph should use a better example than AR Sco and its associated white dwarf pulsars (WDPs) for comparison, because this discussion presents an inaccurate picture of MCVs and IPs in particular. See Stanway et al. 2018, Barrett et al. 2025.

In 125: The paper asserts that J1745 is an LPT, but does not describe the properties of LPTs to show that it is of this subclass. This discussion should be clarified to show that it belongs to this class of radio sources. As the paper discusses in

the conclusions.

ln 129: As in the previous paragraph, the description of radio emitting CVs presents an inaccurate picture of the current situation. Yes, LPTs are bright radio sources, but so is the WDP AR Sco and the magnetic propeller AE Aqr. Both WDPs and magnetic propellers show periodic radio emission. As for polars and typical IPs, the highly polarized radio emission from previous radio surveys are from short observations at higher frequencies (2-10 GHz). Although such observations do not show unambiguously periodic emission, it was been suspected. Therefore, this discussion should be clarified to accurately describe the current state of knowledge. See Barrett et al. 2020.

ln 134: CV orbital periods are known to have a minimum at about 82 minutes (1.36 hours). This is due to the donor star becoming degenerate. The orbital period then increases, but mass transfer does not necessarily cease. This discussion should be modified to clarify this evolutionary scenario. See e.g., Knigge et al 2011.

ln 146: I do not believe this is correct for AR Sco. AR Sco shows pulsed synchrotron emission at the spin and beat period of the WD and possibly plasma and ECME at the orbital and beat periods. This radio emission is mostly emitted throughout the orbital period. This should be clarified. Otherwise it gives an inaccurate description of the star. See Barrett & Gurwell 2025, Stanway et al. 2017.

ln 159: The paper discusses the observed frequency modulations and asserts that they are due to "local plasma acting as an interference screen to the beamed radio emission." There is another simpler explanation that the observed emission is due to the detection gyrosynchrotron emission of low harmonic number (1-6). This also naturally explains the observed elliptical polarization. The detection of these low order harmonics enables the physical parameters of the emitting plasma to be estimated. See White, S et al. 2011. Section 2.4 and Figure 2.1. Gyrosynchrotron emission should be discussed a possible emission mechanism.

ln 185: AR Sco and J1912 are white dwarf pulsars, which means that the WDs are not accreting. The synchrotron emission from AR Sco is caused by relativistic electrons in an ~40 G magnetic field at the spin period of the WD. As previously noted, this discussion should be clarified as it does not give an accurate depiction of these two radio sources. See Barrett & Gurwell 2025.

ln 199: It is recommended that the discussion about the X-ray and radio luminosities follow the discussion about the distance to J1745, i.e., the following paragraph. Asserting a distance to J1745 without a detailed discussion of its estimated distance is rather presumptuous and should be corrected.

ln 199: It is also recommended that the discussion about the X-ray and radio luminosities be reevaluated in light of the distance being an order of magnitude less than the asserted distance and the luminosities being two orders of magnitude less. These smaller values are more in line with similar radio emitting MCVs.

ln 213: The discussion of the distance to J1745 can be considerably improved. First, the orbital period of about 1.35 hours constrains the mass of the companion star to a <0.15 Mo or a spectral type of about M9. See the discussion in the Observations section for details.

ln 303: The paper describes simple modeling of the radio emission and asserts that it is caused by asynchronous rotation of the WD. This is an accurate assessment of the situation. However, the paper does not state than the system is an asynchronous polar, i.e., a polar where the WD's rotation period is a few percent faster than the orbital period. The paper should discuss this scenario.

Observations:

ln 392: The Gaia section identifies J1745 with the star Gaia 5946454415417964032, which seems reasonable. However, the section does not give the Gaia geometric distance of 570 +/- 300 pc. Instead, it proceeds to determine the distance to J1745 using the "Bailer-Jones" geophotometric method and determines a distance of 6.5 +-2.6/1.1 kpc. This distance is unusually large for a CV with such a low mass companion of 0.15 Mo. It also implies an unusually high space velocity of ~150 km/s assuming the measured Gaia proper motion of 4.8 mas/yr. Stars with this velocity are considered hypervelocity stars. See Tutukov et al. 2021. On the other hand, the star has a reasonable space velocity of ~12 km/s for a distance of 570 pc, which is consistent with the barycentric velocity of 15.8 km/s and is characteristic of stars within the solar neighborhood. Its apparent magnitude is also reasonable and similar to other CVs with similar orbital periods. Therefore, the distance to J1745 is likely to be 570 pc and not 6.5 kpc. The result is that most of the arguments about J1745 are incorrect and should be reanalyzed.

Analysis:

ln 496: It is not clear to this reviewer that this section is needed given that Gaia source 4032 is the closest optical source to the radio position and its optical, infrared, and ultraviolet magnitudes are consistent with a magnetic CV.

ln 631: The radio observations described in this paragraph are more naturally associated with gyrosynchrotron emission of low harmonic number (1-6) as it naturally explains the frequency modulated radio emission and elliptical polarization. This possible emission mechanism should be discussed.

ln 650: Note that the error on the orbital frequency is given approximately by the inverse of the product of the length of the observation and the signal-to-noise ratio. For a two year observation and a S/N ratio of 10, this gives an orbital period error of about 0.037 s. See Bretthorst 1998, https://bayes.wustl.edu/glb/book.pdf.

Modelling:

ln 701: Using the revised distance to J1745, the brightness temperature is ~10^12 K. The brightness temperature still requires a coherent emission mechanism, but does not require electron cyclotron maser emission. This brightness temperature is consistent with gyrosynchrotron emission, which naturally explains the elliptical polarization and the presence of broad emission lines at about 1 GHz. Such broad emission lines are often seen in the post-shock region of polars with magnetic fields of a few tens of MG. The emission lines at ~1 GHz imply a magnetic field of ~100 G, a low electron density, and small emission region. Gyrosynchrotron emission does not require the presence of relativistic electrons. A thermal plasma of ~10 keV will suffice. It is recommended that the authors re-evaluate their assertion that ECME is the mostly likely radiation mechanism and include a discussion of gyroshynchrotron emission.

ln 719: The proposed emission model as shown in the Extended Data Figure 1 is rather simplistic and does not include the complexities associated with the propagation of polarized radiation in a magnetized plasma. No mention or estimate of the magnetic field strength nor the plasma density is discussed. Note that the plasma frequency must be less than the gyrofrequency for ECME to escape the plasma. The manuscript does not discuss this issue and show that the plasma density is sufficiently low for ECME to be feasible. Note that the plasma density must be <10^12 electron/cm^3. This is the not the case for gyrosynchrotron emission. These issues need to be discussed.

Supplementary Methods:

ln 1120: The data was fit with a power-law of 2.68, but the paper contains no further discussion of this result and how it may inform the authors about the emission mechanism.

Minor Issues

ln 94: The apparent optical magnitude should be given instead of the absolute magnitude. The absolute magnitude should be deferred to the Methods section.

ln 106: Polar orbital periods range from 1.3 to >4 hours, not <2 hours.

ln 109: Intermediate polar (IP) orbital periods range from 1.3 to >12 hours depending on your definition, not >3 hours.

ln 111: Change "nova" -> "nova outburst". A nova is defined as any CV that has gone through at least one outburst.

Reviewer #2

(Comments for the Author)
The authors present the discovery of a long-period radio transient (LPT), ASKAP J174508.9−505149, in the Rapid ASKAP Continuum Survey (RACS), that appears associated with a cataclysmic variable (CV). This discovery expands upon the emerging field of LPT research by providing the first evidence LPTs may be associated with accreting systems. The authors provide a thorough investigation of J1745, exploring its radio, optical, UV, and X-ray properties with follow-up and archival observations. In doing so, they clearly demonstrate the source’s periodicity and sufficiently support its classification as a CV.

Below, I list a few questions about their methodology and some minor comments on clarity and context. I also include a suggested reference, but the references that are included seem appropriate.

Line 104
I would change this to specify the spectral type (M to K) as not all MS stars can be part of a CV.

Line 114
I hesitate to use AR Sco as a point of comparison, especially with the absence of a seconds-long spin period in J1745. AR Sco is a unique system rather than a typical IP, but I think it is sufficient for the authors to simply note this. After all, there are not many points of comparison given the low number of radio IPs in the literature.

Line 139
I think this second P_orb should be renamed to P_pulse or something similar to avoid confusion.

Line 156
The authors reference Supplementary Table 1, but I could not find this in the submitted manuscript. It is also included in Line 615.

Line 192

It was somewhat difficult to judge the content of this paragraph without a corresponding light curve. Perhaps it would be good to reference Fig. 2 here. I would also like to see the significance the offset between the X-ray emission and the radial velocity posterior, ATCA, and ASKAP pulses discussed at some point as well. I believe it is noted in the Methods, but I think it deserves mentioning in the main body of the article as well.

Line 193
When stating the radio pulses and X-ray emission have the same period, it would be more appropriate to include the fitted period with error bars, in my opinion.

Line 206
Reference 35 is now published, so the reference section should be updated.

Line 267
I suggest adding a reference to Qu & Zhang (2025) on the subject of relativistic ECME:
https://scixplorer.org/abs/2025ApJ...981...34Q/abstract

Line 274
It is unclear what the authors mean by “energises field lines in Jupiter’s magnetosphere.” I suggest rewriting this so that the physics is clearer.

Line 281
Because there is an estimate on the density, I suggest calculating the magnetic field strength in the emission region for ECME using the condition that f_pe < f_ce and compare this value to the possible emission regions in J1745.

Line 290
Because the authors discuss Balmer line emission, it seemed as though they were suggesting ECME was being generated in the disk itself or possibly the accretion column. This was expanded upon in the Methods, but I suggest making this clearer here because this is an important aspect the radio emission.

Line 317
It seems a bit hasty to conclude that the emission is tied to magnetically-driven accretion in this case. Perhaps I don’t quite understand their line of thinking, but it seemed like an open question based on their data whether there was a concrete connection between an increased or decreased accretion rate and the radio pulses. Because the pulse period is so closely correlated to the orbital period, I would have liked the authors to consider rotationally (also orbitally, since the donor is tidally locked) modulated ECME caused by corotation breakdown in the donor star’s magnetosphere (e.g. the discussion of the dMe, UV Cet, in Villadsen & Hallinan 2019: https://scixplorer.org/abs/2019ApJ...871..214V/abstract). Regardless of whether the emission is accretion-driven or not, I feel I should stress that these results are clearly impactful and I commend the authors for the completeness of their work.

Line 570
The authors reference Supplementary Table 2, but I could not find this in the submitted manuscript. It is also included in Line 1166.

Line 623
It would be helpful to include the drift rate as a point of comparison to the MKT observations listed below and other radio bursts.

Line 697
Assuming the size of the emission region to be one solar radius does not seem very conservative. Is it physically possible for the emission region to be that large? I would rather the authors make an estimate of the size of the emission region and support their claim before estimating the brightness temperature.

Line 714
The authors should comment here about the density restrictions. ECME requires a diffuse plasma environment to be produced, which may not be possible in the accretion stream.

Line 724–730
After reviewing Fig. 3 and Extended Data Figure 7, I am wondering if the model can explain why the spacing between pulse pairs seems to increase (hour 2 to 10, bottom panel, Fig. 3) rather than slowly decrease, as shown in Extended Data Figure 7. Perhaps showing one or two more example simulations in the same figure would help illustrate this.

Fig. 2
The graphic on the bottom panel ended up confusing me at first when it came to the inclination of the system. It may be helpful to remind the reader that the system is nearly face-on in the figure caption.

Fig. 3, 4
For the light curves shown in these figures, I would prefer that the authors add error bars to their data points, or include a representative size of the error somewhere in the figure. Given the size of the error bars in Extended Data Table 1, the error bars should be visible, but if they are too small to be useful to the reader, then it is fine to omit them.

Extended Data Figure 2
The authors say in the caption that the pulse eventually returns to the original polarization state. It is unclear how the polarization state is the same when examining the purple and yellow data points. While I am not used to using a Poincare sphere for analysis, it seems as though the pulse migrated 10–20 degrees outward during the time shaded in grey.

Extended Data Figure 7
The last sentence of the caption says that the “model reproduces several key features” of the data. I would like the authors to reiterate which features the readers should look out for in the caption.

Line 1125
Since the authors quote a 3-sigma threshold here, I would like to confirm that they are using a known position for the object (e.g. from Gaia) so that this threshold is appropriate. If there is any offset between the fitted position and known position, this should be stated as well.

Reviewer #3

(Comments for the Author)
The paper describes the detection of a new long period radio transient (LPT). Importantly, the authors use multi-wavelength data to definitively show (for the first time) that this LPT is a cataclysmic variable. This is a highly impactful result, as it establishes that some of the LPT population are accreting white dwarfs. For this reason, it meets the journal's impact criteria. This is a thorough and carefully conducted analysis, and most of my comments are minor. I would like to see the revised version.

1) The blackbody temperature derived from the spectral peak at shorter wavelengths (26,641 K) would be very hot for a white dwarf in a CV near the period gap (e.g. see Pala et al. 2017). This should be mentioned and addressed.

2) Supplementary Tables 1 and 2 are not in the version of the paper that I received. This is confusing, as supplementary tables 3,4 and 5 are there.

3) Second paragraph of the 'Time of Arrival Analysis' section in the Supplementary Methods section: The methodology is a little unclear. A few more details should be given, and all the acronyms should be defined.

4) line 133/4: Barret et al. 2020 do claim that 75% of the detected magnetic CVs show highly circularly polarised radio emission. However, considering the uncertainties on the polarization fractions and fluxes (Table 1 in that paper) and the fact that the variability timescales were not constrained, I don't think that claim is justified. Some magnetic CVs do show this behaviour, but it's not 75% of the population. As this is an important distinction when comparing to the LPT radio emission, consider the statistics in that paper. If you agree with me, then rephrase the statement that '75% of known magnetic CVs' show highly circularly polarised radio emission.

5) line 114: AR Sco is not a prototypical radio-emitting polar. It is a white dwarf pulsar and is spin powered, not accretion powered. The broadband pulsing synchrotron emission seen in AR Sco is also unlike that of the other polars.

6) line 250-254: As there is accretion in this system, the plasma density should be too high for the unipolar induction model to apply.

7) line 647: How was the uncertainty on the radio period determined?

8) Extended Data Figure 6: It's clear why a test companion mass of 0.1 is indicated, but why are masses of 0.05 and 0.15 used?

9) line 102: Polars and intermediate polars are magnetic CVs, so the phrasing that strong HeII lines are consistent with magnetic CVs and the flat spectra and narrow Balmer lines are consistent with polars and intermediate polars is confusing.

10) line 125: 'The observed LPT-like radio emission and polar-like spectral features of ASKAP J1745-5051...' As it was not possible to distinguish whether ASKAP J1745-5051 is a polar or an intermediate polar, it would be best to call it 'magnetic-like'.

11) line 130: Hewitt et al. 2020, Koerding et al. 2008 and Kersten et al. 2025 should also be cited for radio CV detections.

12) line 137: The period minimum in CVs has been detected and confirmed (Gaensicke et al. 2009).

13) line 147: Stanway et al. 2018 should also be cited for the AR Sco radio behaviour

14) line 520: The text refers to narrow spectral features due to potential cosmic rays at ~8300 and ~9800 angstroms in extended data figure 1, but the plot doesn't extend to these wavelengths.

15) Minor comments on figures:
15.1) Some of the text in the following figures is too small: Figure 3 (legend), extended data figure 2, extended data figure 3,

extended data figure 5, extended data figure 9
15.2) In figure 1, it would be good to briefly state in the caption that the highest frequency range of the 0.81 phase spectrum is likely a calibration error. This is mentioned in the main text, but some readers will miss this.
15.3) In figure 1 and extended data figure 1, change 'phases' to 'orbital phases' so that it is clearer for the reader.
15.4) In extended data figure 3 the grey inclined lines and their legends are very faint and and too small to read.
15.5) In the caption of extended data figure 3, it should be specified that the horizontal line is the mean flux for isolated M dwarfs.
15.6) The details and text in the lower panel of figure 2 are too faint and small to be legible.
15.7) The dotted orange line in extended data figure 9 is very difficult to see.
15.8) In figure 2, do the light shaded regions represent the pulse width? If so, this should be stated in the caption.
15.9) In figure 4, labelling these CVs as 'Ridder CVs' is misleading. That paper detailed the detection of 2 new CVs and made a compilation of radio-detected CVs from other papers. It would be more appropriate to give a very short description of the sample (e.g. radio-detected CVs with detected X-ray emission) and then citing Ridder et al. and the original detection papers for this sample.

16) Typos and minor formatting issues:
16.1) line 211: 'has estimated' -> 'has an estimated'
16.2) line 300: 'hinting a white dwarf' -> 'hinting at a white dwarf'
16.3) line 344: 'We obtain 5sigma' -> 'We obtain a 5sigma'
16.4) line 434: There is red text
16.5) line 762: Unfinished grant number
16.6) figure 4 caption: 'correspond to calibration' -> 'corresponds to calibration'
16.7) extended data figure 7: 'Lower panels show the' -> 'The lower panel shows the'
16.8) line 1168: There is blue text
16.9) line 1185: There is a space missing in F_{fit}is
16.10) line 1225: the the
16.11) line 1245: dwarfs's

Version 1:

Reviewer comments:

Reviewer #1

(Comments for the Author)
The author's response has not addressed any of my major criticisms.

First, the response has not fully addressed my comments about the distance to the source. Their distance is clearly incorrect in my opinion and is based on weak arguments. Just because the error on the distance is large does not make the distance incorrect. A more compelling argument needs to be made. In addition, the author did not address my comment about the space velocity of the source. At a distance of ~5700 pc, the velocity of the source is hypersonic, which I strongly doubt is the case. Whereas a distance of ~570 pc gives a reasonable space velocity that is consistent with the average space velocity of stars in the galaxy.

Second, the author has not adequately addressed the issue of gyrosynchrotron emission as a possible emission mechanism. This is a viable emission mechanism if the binary is at a distance of ~570 pc. However, to dismiss gyrosynchrotron emission by saying that such emission has not been detected in other MCVs at that frequency is not a satisfactory response. Like the above comment, a more compelling argument needs to be made. Note that this is an opportunity for the author to show that gyrosynchrotron emission is a viable option for the low frequency radio emission from LPTs and MCVS, and to get recognition for that fact. I know that I surely would.

Reviewer #2

(Comments for the Author)
The authors have addressed the majority of this reviewer’s comments in a satisfactory manner. I have one remaining point that requires clarification.

In response to this reviewer’s comments, the authors provided clarification of their conclusions in Line 277. After reading this addition, the logic appears contradictory. They correctly point out that linear polarization has not yet been detected in CVs and go on to say that the WD that is likely in ASKAP J1745 must therefore be a driver for the production of the observed pulsed emission with the implication that this is due to the presence of linear polarization. CVs also contain a WD, so this does not seem to support their assertion. If this is not the authors’ intended meaning, I suggest they rewrite this section.

Reviewer #3

(Comments for the Author)
The authors have addressed all my concerns. I only have three minor follow-up comments. I don't need to see the manuscript again, and I recommend it for publication.

1) line 114-116: Although it has been suggested that white dwarf pulsars (WDPs) are progenitors to LPTs, Castro Segura et al. 2025 argue against this. The white dwarfs in WDPs are hot, which indicates that they were heated via accretion prior to the WDP phase. The white dwarfs in LPTs are too cool be a further evolutionary stage of WDPs. This should be mentioned.

2) There are 3 typos in the section on plasma frequency in the supplementary methods section: 1) It should be nu_p<nu_B. 2) the plasma frequency is proportional to the square root of the electron density. 3) 'region, which is consistent...'

3) line 1368: 'To calculate calculate...'

Version 2:

Reviewer comments:

Reviewer #1

(Comments for the Author)
Dear Authors,

The paper is much improved, but there is still one important issue that still needs to be addressed before it is accepted for publication.

Major issues:

1. To say that the distance can be as far as 9.1 kpc is unphysical. It is not possible for an M6 dwarf to have an infrared magnitude of ~17.5 mag, or even <20 mag, at a such a distance. As Bailey 1981, Berriman et al. 1985, and Berriman 1987 have shown. The K band surface brightness of K and M stars is weakly dependent on spectral type. In addition, the K magnitude is not significantly affected by interstellar extinction. As a result, the distance depends on three measurements: the K band magnitude K, the radius of the red star R_RD, and the K surface brightness parameter S_K, via the equation:

d = 10^(1 + K/5 + log10(R_RD/R_Sun) - S_K/5).

Your analysis provides values for the V and K band magnitudes and the stellar radius of 21.0, 17.6 and 0.13 R_Sun, resp., Therefore, it is only necessary to estimate S_K. For a V - K of 3.4, Bailey's approximation gives S_K = 4.29 and Berriman's gives 4.21, resulting in distances of 597 and 619 pc, resp. For a distance of >1 kpc, the S_K would need to be as low 3.15 or the K magnitude >18.0. Both of which are highly discrepant from your observations and previous analyses. Therefore, the distance to the ASKAP J1745-5051 is unlikely to be >1 kpc. The authors are encouraged to use the 1 kpc upper limit in the paper.

As a result, the discussions quoting a distance of 9.1 kpc should be changed to 1 kpc, namely lines 213 and 446, and extended data figures 3 and 4 should be adjusted to show this change. The discussion about the Bailer-Jones photogeometric distance is also clearly incorrect based on this discussion and should be removed.

Note that a measurement of the K band magnitude should settle this issue.

2. The paper adequately addresses the issue of gyrosynchrotron emission.

Minor issues:

1. Ln 221: The paper emphasizes that the radio luminosity from J1745-5051 is ~100 greater than 99% of CVs, but almost all of those observations are at higher frequencies and are caused by the electron cyclotron maser instability (ECMI). Whereas, the radio emission from J1745-5051 is unlikely to be caused by relativistic ECMI (see Comment 1). Therefore, what is the point of this statement and how does it add to our understanding of this source?

2. Ln 735: This section should list the observed dispersion measure or its upper limit.

3. Ln 785: The paper should include an estimate of the radio luminosity from the low density plasma (< 10^11 cm^-3) to support their assertion that ECMI from such a low density plasma can produce the observed luminosity.

Comments:

1. Ln 282 & 769: Note that paper [45] proposing relativistic ECMI is almost certainly incorrect, because ECMI requires a non-thermal velocity distribution that is truncated at high energies. Therefore, it is unlikely that a relativistic population of electrons can be generated with such characteristics as the synchrotron timescale is much shorter than the ECMI timescale.

Open Access This Peer Review File is licensed under a Creative Commons Attribution 4.0 International License, which permits use, sharing, adaptation, distribution and reproduction in any medium or format, as long as you give appropriate credit to the original author(s) and the source, provide a link to the Creative Commons license, and indicate if changes were made.
In cases where reviewers are anonymous, credit should be given to 'Anonymous Referee' and the source.
The images or other third party material in this Peer Review File are included in the article's Creative Commons license, unless indicated otherwise in a credit line to the material. If material is not included in the article's Creative Commons license and your intended use is not permitted by statutory regulation or exceeds the permitted use, you will need to obtain permission directly from the copyright holder.
To view a copy of this license, visit https://creativecommons.org/licenses/by/4.0/

Please find attached our revised submission of “Periodic Radio and X-ray Emission from an Accreting White Dwarf Binary” for publication in *Nature Astronomy*. We have highlighted changes in magenta.

We thank the reviewers for their consideration and detailed feedback, which we have taken into account in the revised manuscript. In addition to various minor comments, we focus on a) Reviewer #1’s comments on the source distance and emission mechanism; and b) the accretion/plasma properties and emission regions mentioned in all referees’ comments.

a) Source Distance & Emission Mechanism:
A detailed discussion of the estimated distance is present in the Methods. In response to a number of comments raised on the topic of distance we have prepared a single, detailed response for reference. We first summarise the points regarding the choice of distance (as described in the Methods). Then we clarify the validity of our luminosity and emission arguments in the context of distance.

1. The Gaia parallax distance is unreliable. The parallax_error/parallax = 0.91/1.75 =0.5 meaning that the parallax is of very low significance.
2. This parallax_error/parallax = 0.5 creates a significant bias in the uncertainties towards much larger distances (see Bailer-Jones 2015, Figure 1), resulting in a distance range of approximately 0.4--2 kpc.
3. As noted in the Methods section, the Bailer-Jones photogeometric distances have been found to be more reliable in instances of 0.1<parallax_error/parallax<1 (see Bailer-Jones et al. 2021, Figures 11 & 12) and therefore, combining the parallax and Bailer-Jones photogeometric distance 6.5 (+2.6/-1.1) kpc, we adopt the combined range 0.4--9.1 kpc.
    a. We have updated the luminosity ranges, colour-magnitude diagram, and luminosity plot accordingly.
4. The low significance 570 pc Gaia parallax distance gives a radio luminosity of ~9e18 erg/Hz/s (using the RACS-mid flux). This is nearly two orders of magnitude above even the most luminous of mCVs.
5. Similarly, at 570 pc the brightness temperature lower limit is still >1e13 K, i.e. above the 1e12 K Compton catastrophe limit and therefore requiring a coherent emission mechanism.

We also note that gyrosynchrotron emission is not a physically viable explanation for the observed emission because it cannot produce emission with brightness temperatures in excess of 1e12 K and because, as far as we are not aware, there are no papers that demonstrate elliptically polarised gyrosynchrotron emission at ~GHz frequencies with linear polarisation reaching the levels we observe from ASKAP J1745. We have clarified this in the Emission Mechanism section of the Methods.

b) Accretion/Plasma Properties and Emission Regions:

We have provided clarifications of our interpretation and model in the Main and provided further details in the Methods and Supplementary Methods sections. We discuss how the radio emission is likely coming from a separate, lower density region of the system than the accretion emission (X-ray and spectroscopy). We include additional discussion and calculations relating to the periodicity and phase of the X-ray emission but note that a separate, in-depth exploration of the X-ray properties and plasma physics of this system each warrant their own follow-up paper.

Below we provide point-by-point responses to each of the comments, separated by reviewer. We trust that our updates are satisfactory to the reviewers, and that we have assuaged all major concerns.

Reviewer #1 (Comments for the Author):

Summary

This manuscript presents interesting radio observations of a magnetic cataclysmic variable and should be published, although not in its current form and without major modifications. In this reviewer's opinion, the manuscript contains many inaccuracies, misrepresentations, and incorrect analyses, which should be addressed as specifically noted in the major and minor issues sections below. Note that due to the number of such issues, it is likely that the reviewer has not identified all of them.

In summary, the manuscript asserts that J1745 is a long period radio transient (LPRT), but does not define their characteristics to show that it is. It also asserts that the radio source is at a distance of 5.6 kpc, when the Gaia distance is an order of magnitude less at 570 pc. The nearer distance gives a more reasonable explanation for all of the observations, such as its space velocity and radio, optical, ultraviolet, and X-ray flux. The manuscript then asserts that electron cyclotron maser emission is the cause of the radio emission because of the high brightness temperature, when gyrosynchrotron emission of low harmonic number provides a more natural explanation of the coherent elliptically polarized emission. Finally, the manuscript asserts that J1745 is similar to the magnetic white dwarfs AR Sco, J1912, and J2306, which are non-accreting magnetic cataclysmic variables, whereas J1745 shows none of their observed radio or optical characteristics and does not have a rapidly rotating WD. The manuscript does conclude that J1745 is a magnetic cataclysmic variable with an asynchronously rotating WD, but does not explicitly state that it is likely an asynchronous polar.

>>> We thank the reviewer for their careful reading and extensive comments on our manuscript, which we have endeavoured to take carefully into account for our revised submission. We are grateful for the reviewer's recognition of the uniqueness of this discovery and its importance in understanding the link between cataclysmic variables and the physics of long period radio transients.

Major Issues

In 111: Asynchronous polars usually have spin periods within a few percent of the orbital period and synchronize within a few decades. However, a few of them have larger asychronisms of ~15%. They do not have asynchronisms >20%. They would then be considered IPs. See e.g., Littlefield et al. 2023, ApJ 165, 43 and Littlefield et al. 2023, 943, L24. This discussion should be corrected.
>>> We have reworded the sentence to avoid confusion and clarify that the usual spin periods are "a few percent" faster than the orbital period, and made the reference to the largest asychronicities " up to ~20%" rather than just "up to similar to 20%"; as reported by Halpern et al. 2022 and Rawat et al. 2022.

In 114: AR Sco is not a typical IP. Typical IPs have spin periods of ~10% of the orbital period. See Warner 1995. AR Sco belongs to a subclass of rapidly-rotating magnetic cataclysmic variables (MCVs) that are non-accreting. Its strong synchrotron-powered radio emission is

unusual for MCVs, unlike that of J1745. Most IPs show radio emission similar to polars, i.e., highly circularly polarized emission due to plasma emission or electron cyclotron maser emission (ECME). Therefore, this paragraph should use a better example than AR Sco and its associated white dwarf pulsars (WDPs) for comparison, because this discussion presents an inaccurate picture of MCVs and IPs in particular. See Stanway et al. 2018, Barrett et al. 2025.
>>> We thank the reviewer for pointing this out and have rephrased the sentence to note AR Sco's unique nature. We have also provided context for AR Sco as a possible progenitor class for polar CVs. We do not attempt to generalise the discussion given the low number of known radio-bright intermediate polars.

In 125: The paper asserts that J1745 is an LPT, but does not describe the properties of LPTs to show that it is of this subclass. This discussion should be clarified to show that it belongs to this class of radio sources. As the paper discusses in the conclusions.
>>> We have added a sentence here to explicitly restate these properties.

In 129: As in the previous paragraph, the description of radio emitting CVs presents an inaccurate picture of the current situation. Yes, LPTs are bright radio sources, but so is the WDP AR Sco and the magnetic propeller AE Aqr. Both WDPs and magnetic propellers show periodic radio emission. As for polars and typical IPs, the highly polarized radio emission from previous radio surveys are from short observations at higher frequencies (2-10 GHz). Although such observations do not show unambiguously periodic emission, it was been suspected. Therefore, this discussion should be clarified to accurately describe the current state of knowledge. See Barrett et al. 2020.
>>> Both AR Sco and AE Aqr are only bright radio sources in comparison to other radio-emitting CVs. Compared to most LPTs, all CVs (including AR Sco and AE Aqr) are orders of magnitude fainter in terms of radio luminosity. We are not aware of any confirmed periodic radio emission from magnetic propellers, nor could we find any such references in the literature. We have clarified this in the text.

In 134: CV orbital periods are known to have a minimum at about 82 minutes (1.36 hours). This is due to the donor star becoming degenerate. The orbital period then increases, but mass transfer does not necessarily cease. This discussion should be modified to clarify this evolutionary scenario. See e.g., Knigge et al 2011.
>>> We have replaced the phrase "accretion-stripped" with "low-mass" to avoid making any specific claims. We do not include a deeper discussion of different evolutionary scenarios as this is not within the scope of this work.

In 146: I do not believe this is correct for AR Sco. AR Sco shows pulsed synchrotron emission at the spin and beat period of the WD and possibly plasma and ECME at the orbital and beat periods. This radio emission is mostly emitted throughout the orbital period. This should be clarified. Otherwise it gives an inaccurate description of the star. See Barrett & Gurwell 2025, Stanway et al. 2017.
>>> We thank the reviewer for pointing this out and have clarified that both AR Sco and ILT J1101 have "radio lightcurves that peak around orbital conjunction", as demonstrated by both Marcote et. 2017 (Figure 2) and Stanway et al. 2018 (Figure 4).

In 159: The paper discusses the observed frequency modulations and asserts that they are due to "local plasma acting as an interference screen to the beamed radio emission." There is another simpler explanation that the observed emission is due to the detection gyrosynchrotron emission of low harmonic number (1-6). This also naturally explains the observed elliptical polarization. The detection of these low order harmonics enables the physical parameters of the emitting plasma to be estimated. See White, S et al. 2011. Section 2.4 and Figure 2.1. Gyrosynchrotron emission should be discussed a possible emission mechanism.
>>> Gyrosynchrotron emission is not a physically viable explanation for the observed emission because:
a) We are not aware of any papers that demonstrate elliptically polarised gyrosynchrotron emission with linear polarisation reaching the levels we observe from ASKAP J1745
b) the radio detection exceeds the 10^12 K brightness temperature limit (even at the lower limit Gaia parallax distance of ~0.4 kpc), therefore requiring a coherent emission mechanism (see Melrose & Dulk 1982).

In 185: AR Sco and J1912 are white dwarf pulsars, which means that the WDs are not accreting. The synchrotron emission from AR Sco is caused by relativistic electrons in an ~40 G magnetic field at the spin period of the WD. As previously noted, this discussion should be clarified as it does not give an accurate depiction of these two radio sources. See Barrett & Gurwell 2025.
>>> We do not claim that AR Sco or J1912 are accreting, only that Schwope et al. 2023 have proposed that residual accretion is a plausible explanation for the X-ray variability of J1912.

In 199: It is recommended that the discussion about the X-ray and radio luminosities follow the discussion about the distance to J1745, i.e., the following paragraph. Asserting a distance to J1745 without a detailed discussion of its estimated distance is rather presumptuous and should be corrected.
>>> A detailed discussion of the estimated distance is present in the Methods. In response to a number of comments raised on the topic of distance we have prepared a single, detailed response for reference. We first summarise the points regarding the choice of distance (as described in the Methods). Then we clarify the validity of our luminosity and emission arguments in the context of distance.

1. The Gaia parallax distance is unreliable. The parallax_error/parallax = 0.91/1.75 =0.5 meaning that the parallax is of very low significance.
2. This parallax_error/parallax = 0.5 creates a significant bias in the uncertainties towards much larger distances (see Bailer-Jones 2015, Figure 1), resulting in a distance range of approximately 0.4--2 kpc.
3. As noted in the Methods section, the Bailer-Jones photogeometric distances have been found to be more reliable in instances of 0.1<parallax_error/parallax<1 (see Bailer-Jones et al. 2021, Figures 11 & 12) and) and therefore, combining the parallax and Bailer-Jones photogeometric distance 6.5 (+2.6/-1.1) kpc, we adopt the combined range 0.4--9.1 kpc.
    a. We have updated the luminosity ranges, colour-magnitude diagram, and luminosity plot accordingly.

4. The low significance 570 pc Gaia parallax distance gives a radio luminosity of ~9e18 erg/Hz/s (using the RACS-mid flux). This is nearly two orders of magnitude above even the most luminous of mCVs.
5. Similarly, at 570 pc the brightness temperature lower limit is still >1e13 K, i.e. above the 1e12 K Compton catastrophe limit and therefore requiring a coherent emission mechanism.

In 199: It is also recommended that the discussion about the X-ray and radio luminosities be reevaluated in light of the distance being an order of magnitude less than the asserted distance and the luminosities being two orders of magnitude less. These smaller values are more in line with similar radio emitting MCVs.
>>> Please see the previous response regarding distance and luminosity.

In 213: The discussion of the distance to J1745 can be considerably improved. First, the orbital period of about 1.35 hours constrains the mass of the companion star to a <0.15 Mo or a spectral type of about M9. See the discussion in the Observations section for details.
>>> We discuss this in the Methods section. Please see the previous response regarding the distance.

In 303: The paper describes simple modeling of the radio emission and asserts that it is caused by asynchronous rotation of the WD. This is an accurate assessment of the situation. However, the paper does not state than the system is an asynchronous polar, i.e., a polar where the WD's rotation period is a few percent faster than the orbital period. The paper should discuss this scenario.
>>> We have clarified this point in the text and thank the reviewer for their comment regarding the accuracy of our model. We provided a brief discussion of the scenario in the paragraph in question and described the polar-like characteristics in lines 101, 128 and 219. We leave more detailed discussion to future work.

Observations:

In 392: The Gaia section identifies J1745 with the star Gaia 5946454415417964032, which seems reasonable. However, the section does not give the Gaia geometric distance of 570 +/- 300 pc. Instead, it proceeds to determine the distance to J1745 using the "Bailer-Jones" geophotometric method and determines a distance of 6.5 +-2.6/1.1 kpc. This distance is unusually large for a CV with such a low mass companion of 0.15 Mo. It also implies an unusually high space velocity of ~150 km/s assuming the measured Gaia proper motion of 4.8 mas/yr. Stars with this velocity are considered hypervelocity stars. See Tutukov et al. 2021. On the other hand, the star has a reasonable space velocity of ~12 km/s for a distance of 570 pc, which is consistent with the barycentric velocity of 15.8 km/s and is characteristic of stars within the solar neighborhood. Its apparent magnitude is also reasonable and similar to other CVs with similar orbital periods. Therefore, the distance to J1745 is likely to be 570 pc and not 6.5 kpc. The result is that most of the arguments about J1745 are incorrect and should be reanalyzed.
>>> Please see the previous response regarding distance.

Analysis:

In 496: It is not clear to this reviewer that this section is needed given that Gaia source 4032 is the closest optical source to the radio position and its optical, infrared, and ultraviolet magnitudes are consistent with a magnetic CV.
>>> We prefer to retain this section for the purpose of being more scientifically rigorous. Especially given the various positional uncertainties and respective PSFs at different wavelengths. The purpose of the chance coincidence analysis is to demonstrate whether or not it is reasonable to associate the radio source with this particular optical source.

In 631: The radio observations described in this paragraph are more naturally associated with gyrosynchrotron emission of low harmonic number (1-6) as it naturally explains the frequency modulated radio emission and elliptical polarization. This possible emission mechanism should be discussed.
>>> Please see the previous responses regarding why gyrosynchrotron emission is not a physically viable explanation. It cannot explain the high levels of elliptical polarisation nor the high brightness temperature required by even the closest distance.

In 650: Note that the error on the orbital frequency is given approximately by the inverse of the product of the length of the observation and the signal-to-noise ratio. For a two year observation and a S/N ratio of 10, this gives an orbital period error of about 0.037 s. See Bretthorst 1998, https://bayes.wustl.edu/glb/book.pdf.
>>> We thank the reviewer for pointing out this confusion. We have added some additional context to clarify that the higher accuracy radio period is obtained from Timing of Arrival analysis not from periodogram statistics (the latter is the focus of Bretthorst 1998).

Modelling:

In 701: Using the revised distance to J1745, the brightness temperature is ~10^12 K. The brightness temperature still requires a coherent emission mechanism, but does not require electron cyclotron maser emission. This brightness temperature is consistent with gyrosynchrotron emission, which naturally explains the elliptical polarization and the presence of broad emission lines at about 1 GHz. Such broad emission lines are often seen in the post-shock region of polars with magnetic fields of a few tens of MG. The emission lines at ~1 GHz imply a magnetic field of ~100 G, a low electron density, and small emission region. Gyrosynchrotron emission does not require the presence of relativistic electrons. A thermal plasma of ~10 keV will suffice. It is recommended that the authors re-evaluate their assertion that ECME is the mostly likely radiation mechanism and include a discussion of gyroshynchrotron emission.
>>> Please see the previous responses regarding why gyrosynchrotron emission is not a physically viable explanation. It cannot the observed levels of elliptical polarisation nor the high brightness temperature required.

In 719: The proposed emission model as shown in the Extended Data Figure 1 is rather simplistic and does not include the complexities associated with the propagation of polarized radiation in a magnetized plasma. No mention or estimate of the magnetic field strength nor the plasma density is discussed. Note that the plasma frequency must be less than the gyrofrequency for ECME to escape the plasma. The manuscript does not discuss this issue and show that the plasma density is sufficiently low for ECME to be feasible. Note that the

plasma density must be <10^12 electron/cm^3. This is the not the case for gyrosynchrotron emission. These issues need to be discussed.
>>> As we discuss in the paper, the focus of this work is the discovery and multi-wavelength characterisation of ASKAP J1745. We intentionally provide this as a simplistic geometric model in order to show that it already reproduces many of the observed features, not as an attempt to obtain a complete model; though both the magnetic field strengths and plasma densities are discussed. Extensive simulations and determination of a detailed physical model are beyond the scope of this paper.

Supplementary Methods:

ln 1120: The data was fit with a power-law of 2.68, but the paper contains no further discussion of this result and how it may inform the authors about the emission mechanism.
>>> As noted because of the low SNR, further analysis of the MALS data was not included as it would not have added to this work.

Minor Issues

ln 94: The apparent optical magnitude should be given instead of the absolute magnitude. The absolute magnitude should be deferred to the Methods section.
>>> We thank the reviewer for noting this. We have added the apparent G-band magnitude here and moved the absolute magnitude to the Methods section.

ln 106: Polar orbital periods range from 1.3 to >4 hours, not <2 hours.
>>> We have changed from "P_orb < 2 hr" to "P_orb~1.3--4 hr" to include the full range.

ln 109: Intermediate polar (IP) orbital periods range from 1.3 to >12 hours depending on your definition, not >3 hours.
>>> We have changed from "P_orb > 3 hr" to "P_orb~1.3--12 hr" to include the full range.

ln 111: Change "nova" -> "nova outburst". A nova is defined as any CV that has gone through at least one outburst.
>>> We have fixed this to clarify the point.

Reviewer #2 (Comments for the Author):

The authors present the discovery of a long-period radio transient (LPT), ASKAP J174508.9−505149, in the Rapid ASKAP Continuum Survey (RACS), that appears associated with a cataclysmic variable (CV). This discovery expands upon the emerging field of LPT research by providing the first evidence LPTs may be associated with accreting systems. The authors provide a thorough investigation of J1745, exploring its radio, optical, UV, and X-ray properties with follow-up and archival observations. In doing so, they clearly demonstrate the source’s periodicity and sufficiently support its classification as a CV.

Below, I list a few questions about their methodology and some minor comments on clarity and context. I also include a suggested reference, but the references that are included seem appropriate.

>>> We thank the reviewer for their careful reading and insightful comments on our manuscript, which we have endeavoured to take carefully into account for our revised submission. We are grateful for the reviewer's recognition of the uniqueness of this discovery and its importance in understanding the link between cataclysmic variables and the physics of long period radio transients.

Line 104
I would change this to specify the spectral type (M to K) as not all MS stars can be part of a CV.
>>> We have added "(usually of spectral type K to M)" after the phrase "main-sequence companion" to clarify.

Line 114
I hesitate to use AR Sco as a point of comparison, especially with the absence of a seconds-long spin period in J1745. AR Sco is a unique system rather than a typical IP, but I think it is sufficient for the authors to simply note this. After all, there are not many points of comparison given the low number of radio IPs in the literature.
>>> We thank the reviewer for pointing this out and have rephrased the sentence to note AR Sco's unique nature. We have also provided context for AR Sco as a possible progenitor class for polar CVs.

Line 139
I think this second P_orb should be renamed to P_pulse or something similar to avoid confusion.
>>> We thank the reviewer for this suggestion and have changed references to the radio period from "P_orb" to" P_radio".

Line 156
The authors reference Supplementary Table 1, but I could not find this in the submitted manuscript. It is also included in Line 615.
>>> We apologise to the reviewers for this oversight. Supplementary Table 1 was meant to be provided as a supplementary .csv file. We have included Supplementary Table 1 as an attached file in our submission.

Line 192
It was somewhat difficult to judge the content of this paragraph without a corresponding light curve. Perhaps it would be good to reference Fig. 2 here. I would also like to see the significance the offset between the X-ray emission and the radial velocity posterior, ATCA, and ASKAP pulses discussed at some point as well. I believe it is noted in the Methods, but I think it deserves mentioning in the main body of the article as well.
>>> We have added a reference to Fig. 2. We have also added a sentence that summarises the significance of the offset between the X-ray and the RV posterior/radio pulses; and added a corresponding section in the Supplementary Methods.

Line 193
When stating the radio pulses and X-ray emission have the same period, it would be more appropriate to include the fitted period with error bars, in my opinion.
>>> We have included the X-ray period (P_X) for comparison and updated the Pulse Periodicity section of the Methods accordingly.

Line 206
Reference 35 is now published, so the reference section should be updated.
>>> We have updated this reference to the SRSC. And have similarly updated any other recently published references. Thank you.

Line 267
I suggest adding a reference to Qu & Zhang (2025) on the subject of relativistic ECME: https://scixplorer.org/abs/2025ApJ...981...34Q/abstract
>>> We thank the reviewer for this suggestion and have added a citation to Qu & Zhang (2025).

Line 274
It is unclear what the authors mean by "energises field lines in Jupiter's magnetosphere." I suggest rewriting this so that the physics is clearer.
>>> We thank the reviewer for pointing this out and have reworded for improved clarity.

Line 281
Because there is an estimate on the density, I suggest calculating the magnetic field strength in the emission region for ECME using the condition that f_pe < f_ce and compare this value to the possible emission regions in J1745.
>>> We have clarified the geometry of our model in the Main as well as explained why ~GHz ECME is not likely to be observable close to the WD surface. As such we do not calculate the magnetic field strength from the Balmer emission region. In the Methods modelling section we have added a note about electron density limit in the ECME region inferred from the plasma condition and a corresponding section of the Supplementary Methods.

Line 290
Because the authors discuss Balmer line emission, it seemed as though they were suggesting ECME was being generated in the disk itself or possibly the accretion column. This was expanded upon in the Methods, but I suggest making this clearer here because this is an important aspect the radio emission.
>>> We have added clarification about the ECME likely originating in a lower density region and switched the order of the subsequent paragraphs in order to follow this with an explicit comment about the location of the radio emission in our model.

Line 317
It seems a bit hasty to conclude that the emission is tied to magnetically-driven accretion in this case. Perhaps I don't quite understand their line of thinking, but it seemed like an open question based on their data whether there was a concrete connection between an increased or decreased accretion rate and the radio pulses. Because the pulse period is so closely correlated to the orbital period, I would have liked the authors to consider rotationally (also orbitally, since the donor is tidally locked) modulated ECME caused by corotation

breakdown in the donor star's magnetosphere (e.g. the discussion of the dMe, UV Cet, in Villadsen & Hallinan 2019: https://scixplorer.org/abs/2019ApJ...871..214V/abstract). Regardless of whether the emission is accretion-driven or not, I feel I should stress that these results are clearly impactful and I commend the authors for the completeness of their work.
>>> We thank the reviewer for raising this point of confusion and have clarified the reasoning behind this (line 277). While co-rotation breakdown is common in many other systems, none exhibit the levels of linear polarisation observed in ASKAP J1745 or other LPTs. Similarly, the radio luminosity from ASKAP J1745, even at the lowest distance limit, is at least 2-3 orders of magnitude greater than that observed from rotationally modulated stellar emission

Line 570
The authors reference Supplementary Table 2, but I could not find this in the submitted manuscript. It is also included in Line 1166.
>>> We apologise to the reviewers for this oversight. Supplementary Tables 1 & 2 were meant to be provided as supplementary .csv files. We have imported Supplementary Table 2 into the revised manuscript and included Supplementary Table 1 as an attached file in our submission.

Line 623
It would be helpful to include the drift rate as a point of comparison to the MKT observations listed below and other radio bursts.
>>> We have added the drift rate of the ATCA observation as a point of comparison.

Line 697
Assuming the size of the emission region to be one solar radius does not seem very conservative. Is it physically possible for the emission region to be that large? I would rather the authors make an estimate of the size of the emission region and support their claim before estimating the brightness temperature.
>>> We have no information on the size of the emission region. The purpose here is not to precisely measure the brightness temperature, rather to constrain the lower limit on the brightness temperature in order to require a coherent emission mechanism. A one solar radius emission region is conservative inasmuch as it underestimates the lower limit on the brightness temperature.

Line 714
The authors should comment here about the density restrictions. ECME requires a diffuse plasma environment to be produced, which may not be possible in the accretion stream.
>>> We have added a clarification here about the electron density limits placed by the Balmer ratio and ECME conditions, respectively, and how they indicate a lack of co-spatiality. We provide the corresponding calculations for this in the Supplementary Methods.

Line 724–730
After reviewing Fig. 3 and Extended Data Figure 7, I am wondering if the model can explain why the spacing between pulse pairs seems to increase (hour 2 to 10, bottom panel, Fig. 3) rather than slowly decrease, as shown in Extended Data Figure 7. Perhaps showing one or two more example simulations in the same figure would help illustrate this.

>>> We have added an additional example in Extended Data Figure 7 to help illustrate this point.

Fig. 2
The graphic on the bottom panel ended up confusing me at first when it came to the inclination of the system. It may be helpful to remind the reader that the system is nearly face-on in the figure caption.
>>> We thank the reviewer for pointing out this confusion. We have added a note about the face-on inclination of the graphic in the caption.

Fig. 3, 4
For the light curves shown in these figures, I would prefer that the authors add error bars to their data points, or include a representative size of the error somewhere in the figure. Given the size of the error bars in Extended Data Table 1, the error bars should be visible, but if they are too small to be useful to the reader, then it is fine to omit them.
>>> We thank the reviewer for this suggestion and have updated Figures 3 and 4 to include error bars and made the figures clearer by removing the connecting line between points.

Extended Data Figure 2
The authors say in the caption that the pulse eventually returns to the original polarization state. It is unclear how the polarization state is the same when examining the purple and yellow data points. While I am not used to using a Poincare sphere for analysis, it seems as though the pulse migrated 10–20 degrees outward during the time shaded in grey.
>>> We thank the reviewer for pointing this out and have added a sentence in the caption to clarify that the polarisation state returns to the original position angle.

Extended Data Figure 7
The last sentence of the caption says that the “model reproduces several key features” of the data. I would like the authors to reiterate which features the readers should look out for in the caption.
>>> We thank the reviewer for this suggestion. We have added a the reiteration of features "including: double-peaked pulses, variable spacing been pulse pairs, modulation of frequency cutoffs, and intermittency" to the end of the caption.

Line 1125
Since the authors quote a 3-sigma threshold here, I would like to confirm that they are using a known position for the object (e.g. from Gaia) so that this threshold is appropriate. If there is any offset between the fitted position and known position, this should be stated as well.
>>> We wish to clarify to the reviewer that these are low significance forced fits taken at the position of the ASKAP discovery coordinates.

Reviewer #3 (Comments for the Author):

The paper describes the detection of a new long period radio transient (LPT). Importantly, the authors use multi-wavelength data to definitively show (for the first time) that this LPT is a cataclysmic variable. This is a highly impactful result, as it establishes that some of the LPT population are accreting white dwarfs. For this reason, it meets the journal's impact

criteria. This is a thorough and carefully conducted analysis, and most of my comments are minor. I would like to see the revised version.

>>> We thank the reviewer for their careful reading and insightful comments on our manuscript, which we have endeavoured to take carefully into account for our revised submission. We are grateful for the reviewer's recognition of the uniqueness of this discovery and its importance in understanding the link between cataclysmic variables and the physics of long period radio transients.

1) The blackbody temperature derived from the spectral peak at shorter wavelengths (26,641 K) would be very hot for a white dwarf in a CV near the period gap (e.g. see Pala et al. 2017). This should be mentioned and addressed.
>>> We thank the reviewer for this suggestion. We have added a mention and discussion of this point in the Methods, where the blackbody temperatures are given.

2) Supplementary Tables 1 and 2 are not in the version of the paper that I received. This is confusing, as supplementary tables 3,4 and 5 are there.
>>> We apologise to the reviewers for this oversight. Supplementary Tables 1 & 2 were meant to be provided as supplementary .csv files. We have imported Supplementary Table 2 into the revised manuscript and included Supplementary Table 1 as an attached file in our submission.

3) Second paragraph of the 'Time of Arrival Analysis' section in the Supplementary Methods section: The methodology is a little unclear. A few more details should be given, and all the acronyms should be defined.
>>> We thank the reviewer for raising this issue and have updated this paragraph to clarify the method and its parameters; and provided a relevant reference for further details.

4) line 133/4: Barret et al. 2020 do claim that 75% of the detected magnetic CVs show highly circularly polarised radio emission. However, considering the uncertainties on the polarization fractions and fluxes (Table 1 in that paper) and the fact that the variability timescales were not constrained, I don't think that claim is justified. Some magnetic CVs do show this behaviour, but it's not 75% of the population. As this is an important distinction when comparing to the LPT radio emission, consider the statistics in that paper. If you agree with me, then rephrase the statement that '75% of known magnetic CVs' show highly circularly polarised radio emission.
>>> We thank the reviewer for raising this and have rephrased the state to be "several magnetic CVs". This avoids a potentially misleading claim about the larger population and takes the uncertainties of the polarisation fractions into account (namely that 12/24 reported polarisation fractions are smaller than their respective uncertainties).

5) line 114: AR Sco is not a prototypical radio-emitting polar. It is a white dwarf pulsar and is spin powered, not accretion powered. The broadband pulsing synchrotron emission seen in AR Sco is also unlike that of the other polars.
>>> We thank the reviewer for pointing this out and have rephrased the sentence to note AR Sco's unique nature. We have also provided context for AR Sco as a possible progenitor class for polar CVs.

6) line 250-254: As there is accretion in this system, the plasma density should be too high for the unipolar induction model to apply.
>>> We thank the reviewer for raising this point, though note that we mention the unipolar induction model as a contextual example. While the reviewer is correct to note that coherent emission would be quenched in an accreting region, the presence of accretion in a system does not preclude such a mechanism from emitting outside of the accretion region.

7) line 647: How was the uncertainty on the radio period determined?
>>> We have added a sentence to describe how we use the HWHM from the Lomb-Scargle periodogram; propagated appropriately to the period.

8) Extended Data Figure 6: It's clear why a test companion mass of 0.1 is indicated, but why are masses of 0.05 and 0.15 used?
>>> We included these additional masses to support the point that the system inclination we obtain is not sensitive to the exact mass chosen (line 236)

9) line 102: Polars and intermediate polars are magnetic CVs, so the phrasing that strong HeII lines are consistent with magnetic CVs and the flat spectra and narrow Balmer lines are consistent with polars and intermediate polars is confusing.
>>> We thank the reviewer for pointing out this confusing language and have reworded the sentence for clarity.

10) line 125: 'The observed LPT-like radio emission and polar-like spectral features of ASKAP J1745-5051...' As it was not possible to distinguish whether ASKAP J1745-5051 is a polar or an intermediate polar, it would be best to call it 'magnetic-like'.
>>> We have reworded it to be “magnetic CV-like”. Using "magnetic-like" might be somewhat unclear.

11) line 130: Hewitt et al. 2020, Koerding et al. 2008 and Kersten et al. 2025 should also be cited for radio CV detections.
>>> While these references may be relevant in a broader context we opt not to include them due to formatting limitations (<50 citations) coupled with the fact that our work is not focussed on nova-like or dwarf novae type CVs.

12) line 137: The period minimum in CVs has been detected and confirmed (Gaensicke et al. 2009).
>>> We have replaced the word "proposed" with "shown" and added the Gänsicke et al 2009 reference.

13) line 147: Stanway et al. 2018 should also be cited for the AR Sco radio behaviour
>>> This citation has been added.

14) line 520: The text refers to narrow spectral features due to potential cosmic rays at ~8300 and ~9800 angstroms in extended data figure 1, but the plot doesn't extend to these wavelengths.

>>> We thank the reviewer for pointing this out. A previous version of the plot had extended to these wavelengths, which we do not display due to the lower data quality and lack of features, besides those likely due to cosmic rays. This text has been corrected.

15) Minor comments on figures:
15.1) Some of the text in the following figures is too small: Figure 3 (legend), extended data figure 2, extended data figure 3, extended data figure 5, extended data figure 9
>>> We have enlarged the Fig. 3 legends, and enlarged the text and legend (as necessary) in Fig. 3, Extended Data Figure 2, Extended Data Figure 3, Extended Data Figure 5, and Extended Data Figure 9.

15.2) In figure 1, it would be good to briefly state in the caption that the highest frequency range of the 0.81 phase spectrum is likely a calibration error. This is mentioned in the main text, but some readers will miss this.
>>> We thank the reviewer for this helpful suggestion and have added "The red excess in the $\phi=0.81$ spectrum (second from the top) is likely due to a calibration error." to the caption.

15.3) In figure 1 and extended data figure 1, change 'phases' to 'orbital phases' so that it is clearer for the reader.
>>> We have updated these captions to reflect this for clarity. Thank you.

15.4) In extended data figure 3 the grey inclined lines and their legends are very faint and and too small to read.
>>> We thank the reviewer for this feedback. We have increased the opacity of the lines and labels, and increased the size of the labels themselves.

15.5) In the caption of extended data figure 3, it should be specified that the horizontal line is the mean flux for isolated M dwarfs.
>>> We thank the reviewer for this important suggestion. We have added the word "isolated" in the caption.

15.6) The details and text in the lower panel of figure 2 are too faint and small to be legible.
>>> We have increased the size and opacity of the details (and the panel itself).

15.7) The dotted orange line in extended data figure 9 is very difficult to see.
>>> We have increased the linewidth and decreased the background opacity to improve visibility.

15.8) In figure 2, do the light shaded regions represent the pulse width? If so, this should be stated in the caption.
>>> We have stated this in the caption.

15.9) In figure 4, labelling these CVs as 'Ridder CVs' is misleading. That paper detailed the detection of 2 new CVs and made a compilation of radio-detected CVs from other papers. It would be more appropriate to give a very short description of the sample (e.g. radio-detected

CVs with detected X-ray emission) and then citing Ridder et al. and the original detection papers for this sample.
>>> We have relabelled the CVs in this Figure and updated the caption accordingly.

16) Typos and minor formatting issues:
16.1) line 211: 'has estimated' -> 'has an estimated'
>>> We have fixed this typo. Thank you.

16.2) line 300: 'hinting a white dwarf' -> 'hinting at a white dwarf'
>>> We have fixed this typo. Thank you.

16.3) line 344: 'We obtain 5sigma' -> 'We obtain a 5sigma'
>>> We have fixed this typo, using the plural "limits". Thank you.

16.4) line 434: There is red text
>>> The text colour has been fixed.

16.5) line 762: Unfinished grant number
>>> We thank the reviewer for pointing out this oversight. We have added the missing ASKAP project code (ASXXX -> AS113).

16.6) figure 4 caption: 'correspond to calibration' -> 'corresponds to calibration'
>>> We have fixed this typo. Thank you.

16.7) extended data figure 7: 'Lower panels show the' -> 'The lower panel shows the'
>>> We have left this as plural following the addition of a second panel to demonstrate examples of both rising and falling pulse frequencies.

16.8) line 1168: There is blue text
>>> This blue text is a hyperlink to the documentation being referenced.

16.9) line 1185: There is a space missing in F_{fit}is
>>> We have fixed this typo. Thank you.

16.10) line 1225: the the
>>> We have fixed this typo. Thank you.

16.11) line 1245: dwarfs's
>>> We have changed "dwarfs’s" to "dwarf’s". Thank you.

Dear Reviewers,

- We thank the reviewers for their additional feedback, which we have taken into account in the revised manuscript. In addition to fixing the minor comments from Reviewers #2 and #3, we focus largely on Reviewer #1's concerns regarding the source distance and emission mechanism.

- We note that, as discussed in our previous reviewer response, the brightness temperature of the observed emission requires a coherent emission mechanism, even at the closer 570 pc distance. Consequently, the observed emission cannot be physically explained by a gyrosynchrotron mechanism, which is an incoherent process. We also note that the Gaia parallax distance and space velocity both depend on a poorly constrained and unreliable astrometric solution, and have added a new section to the Supplementary Information detailing this.

- Below we provide point-by-point responses to each of the comments, separated by reviewer. We hope that these changes are satisfactory to the reviewers, and that we have assuaged any remaining concerns.

Reviewer #1 (Comments for the Author):

The author's response has not addressed any of my major criticisms.

>>> We thank the reviewer for their additional close reading of our revised manuscript. We have endeavoured to take these final comments into account for our new submission.

First, the response has not fully addressed my comments about the distance to the source. Their distance is clearly incorrect in my opinion and is based on weak arguments. Just because the error on the distance is large does not make the distance incorrect. A more compelling argument needs to be made. In addition, the author did not address my comment about the space velocity of the source. At a distance of ~5700 pc, the velocity of the source is hypersonic, which I strongly doubt is the case. Whereas a distance of ~570 pc gives a reasonable space velocity that is consistent with the average space velocity of stars in the galaxy.
>>>Throughout our analysis we have used a conservative distance range of 0.4–9.1 kpc for the source.

We summarise our reasoning behind this choice of range below:

- Estimating the distance using the Gaia parallax gives a value of 0.57 kpc (+0.62/-0.19 kpc)
- However, the uncertainty on Gaia parallax and the large `astrometric_excess_noise` (3.84 mas - which is larger than both the parallax and proper motion measurements) mean that this distance is unreliable, so we also use the Bailer-Jones photogeometric method.
- Using this method gives a distance estimate of 6.5 kpc (+2.6/-1.1 kpc)
- At the lower distance 0.57 kpc, the implied space velocity would be ~43 km/s, which as the reviewer notes is consistent with the average space velocity of (thin disk) stars in the Galaxy.
- However, the uncertainties on Gaia parallax, proper motions, and the large `astrometric_excess_noise` mean that this space velocity is unreliable
- At the higher distance 6.5 kpc, the implied space velocity would be ~157 km/s. This is consistent with a thick disk Galactic star. It may fit into the category of a “flying star” or “runaway star”, both ≤ 300 km/s. This is according to the Tutukov et al. 2021 paper that the reviewer previously suggested which, in Section 2.5, defines “hypervelocity stars” as those with a space velocity ≥ 700 km/s.
- Calculating space velocities from these Gaia values with such high uncertainties is unreliable. However, even if we do so, the implied space velocities are reasonable. Hence we do not agree that the implied velocity can be used to rule out the Bailer-Jones distance estimates.

To clarify and address this concern we have made the following changes to the manuscript text:

- Explained the issues with the Gaia data in detail in the Supplementary information.
- Restructured Methods section on Gaia and the distance estimates.

We note that our conclusions are not sensitive to the exact distance adopted and, as such, we choose not to assume a single preferred value. These conclusions include:

- A radio luminosity at least an order of magnitude greater than the most luminous known CV and greater than 99% of radio stars
- An X-ray luminosity consistent with accretion

- A brightness temperature necessitating a coherent emission mechanism.

Instead, we retain the full plausible distance range, including the Bailer-Jones photogeometric estimate, to ensure a conservative interpretation.

Second, the author has not adequately addressed the issue of gyrosynchrotron emission as a possible emission mechanism. This is a viable emission mechanism if the binary is at a distance of ~570 pc. However, to dismiss gyrosynchrotron emission by saying that such emission has not been detected in other MCVs at that frequency is not a satisfactory response. Like the above comment, a more compelling argument needs to be made. Note that this is an opportunity for the author to show that gyrosynchrotron emission is a viable option for the low frequency radio emission from LPTs and MCVS, and to get recognition for that fact. I know that I surely would.
>>> We have added text to the paper which clarifies that some radio emission from CVs may be produced by gyrosynchrotron processes, as proposed by Barrett & Gurwell 2025. Below we provide calculations and a plot which demonstrate that the pulsed emission from ASKAP J1745 (typically ranging from 1–6 mJy/beam but peaking at ~22 mJy/beam in RACS-mid) has a brightness temperature lower limit above 10^12 K for distances down to ~220 pc; noting that the lower limit of the Gaia parallax distance is ~370 pc. This strongly suggests that a coherent emission mechanism is required to produce the observed emission. Consequently, the observed emission cannot be physically explained by a gyrosynchrotron mechanism, which is an incoherent process.

omega = π * arctan(r/d)^2
T_b = F*c^2 / (2*k_b * nu^2 * omega)

We take a typical central frequency nu=1 GHz and flux F=1 mJy. We take the upper limit of a 1 solar radius emission, which makes the brightness temperature T_b a lower limit.

Distance [pc]	Omega	T_b [K]
300	1.77e-20	1.83e12
500	5.39e-21	5.09e12
1000	1.60e-21	2.04e13

For the 5 mJy/beam flux, the brightness temperature is above 10^12 K down to a distance of ~100 pc. For the 20 mJy/beam flux, the brightness temperature is above 10^12 K down to a distance of ~50 pc.

Therefore the pulsed emission that we observe from ASKAP J1745 necessitates a coherent emission mechanism. Gyrosynchrotron is an incoherent emission mechanism. At 570 pc the brightness temperature lower limit is still >1e13 K, i.e. above the 1e12 K Compton catastrophe limit and therefore requiring a coherent emission mechanism.

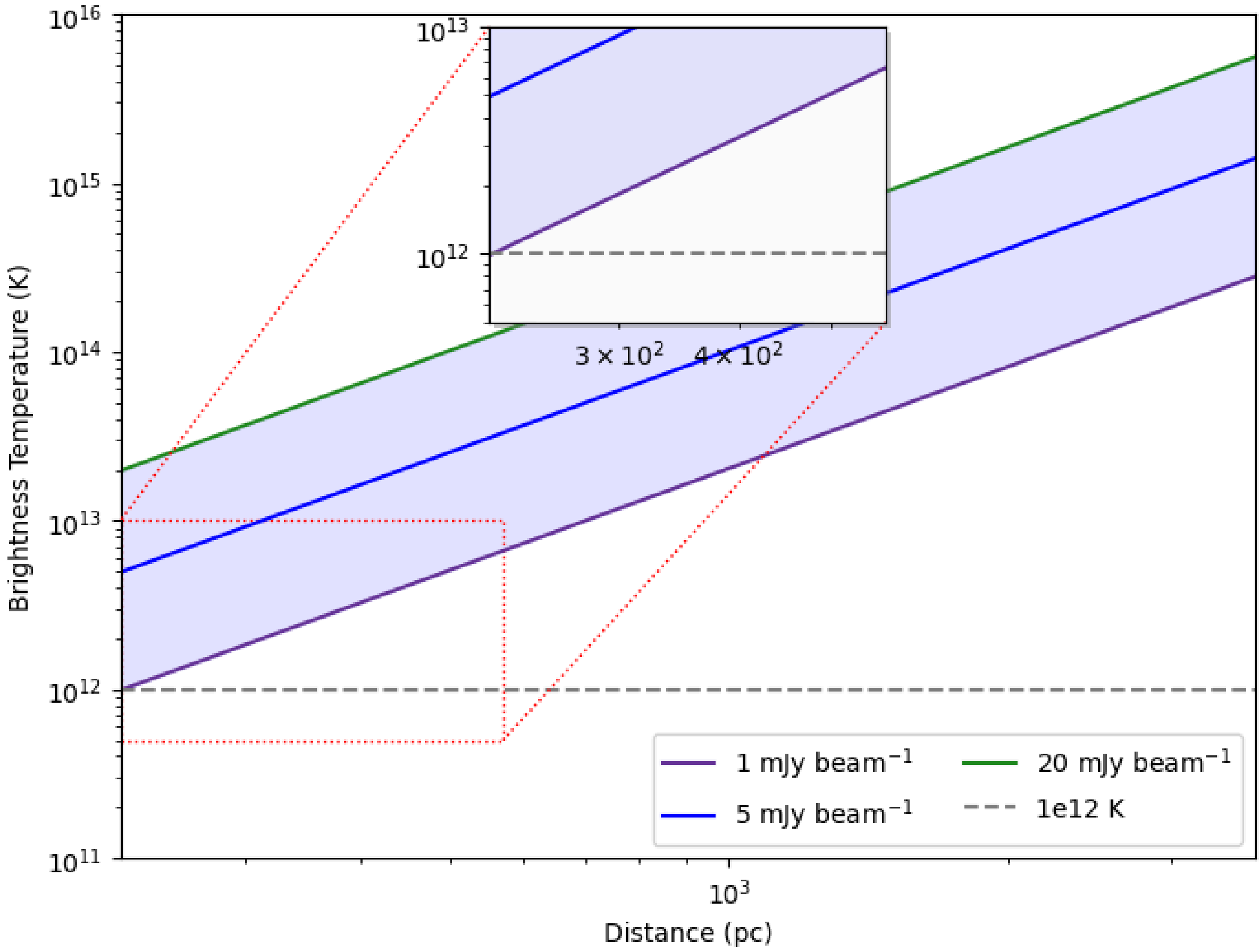

Reviewer #2 (Comments for the Author):

The authors have addressed the majority of this reviewer's comments in a satisfactory manner. I have one remaining point that requires clarification.
>>> We thank the reviewer for their further reading. We have endeavoured to address their remaining comment in our newly revised submission.

In response to this reviewer's comments, the authors provided clarification of their conclusions in Line 277. After reading this addition, the logic appears contradictory. They correctly point out that linear polarization has not yet been detected in CVs and go on to say that the WD that is likely in ASKAP J1745 must therefore be a driver for the production of the observed pulsed emission with the implication that this is due to the presence of linear polarization. CVs also contain a WD, so this does not seem to support their assertion. If this is not the authors' intended meaning, I suggest they rewrite this section.
>>> We have rewritten this paragraph to clarify the logic and hope we have now adequately addressed the original comment.

Reviewer #3 (Comments for the Author):

The authors have addressed all my concerns. I only have three minor follow-up comments. I don't need to see the manuscript again, and I recommend it for publication.

>>> We thank the reviewer for their further reading. We have endeavoured to address their remaining minor comments in our newly revised submission.

1) line 114-116: Although it has been suggested that white dwarf pulsars (WDPs) are progenitors to LPTs, Castro Segura et al. 2025 argue against this. The white dwarfs in WDPs are hot, which indicates that they were heated via accretion prior to the WDP phase. The white dwarfs in LPTs are too cool be a further evolutionary stage of WDPs. This should be mentioned.
>>> We have noted the contrary view of Castro Segura et al. 2025 here. We have also commented on it in the Methods section where we discuss the high WD temperature of ASKAP J1745 in the context of it being an accreting system.

2) There are 3 typos in the section on plasma frequency in the supplementary methods section: 1) It should be nu_p<nu_B. 2) the plasma frequency is proportional to the square root of the electron density. 3) 'region, which is consistent...'
>>> We have fixed these typos accordingly.

3) line 1368: 'To calculate calculate...'
>>> We have fixed this typo.

Please find attached our final revised manuscript of “Periodic Radio and X-ray Emission from an Accreting White Dwarf Binary” for publication in *Nature Astronomy*. We have highlighted new changes from this round in magenta, and removed the colour highlights for changes from previous rounds.

We thank the reviewer for their additional comments, which we have taken into account in the revised manuscript. In addition to addressing these minor comments, we have endeavoured to implement all of the requested editorial and formatting-related changes.

Below we provide point-by-point responses to each of the comments. We hope that this version satisfies the referee’s final requests and the Nature Astronomy guidelines.

We are currently drafting a press release including artwork that we wish to submit for consideration as cover artwork.

Reviewer #1 (Comments for the Author):

Dear Authors,

The paper is much improved, but there is still one important issue that still needs to be addressed before it is accepted for publication.

>>> We thank the reviewer for their further review of our manuscript. We have taken these final comments into account for our new submission.

Major issues:

1. To say that the distance can be as far as 9.1 kpc is unphysical. It is not possible for an M6 dwarf to have an infrared magnitude of ~17.5 mag, or even <20 mag, at a such a distance. As Bailey 1981, Berriman et al. 1985, and Berriman 1987 have shown. The K band surface brightness of K and M stars is weakly dependent on spectral type. In addition, the K magnitude is not significantly affected by interstellar extinction. As a result, the distance depends on three measurements: the K band magnitude K, the radius of the red star R_RD, and the K surface brightness parameter S_K, via the equation:

d = 10^(1 + K/5 + log10(R_RD/R_Sun) - S_K/5).

Your analysis provides values for the V and K band magnitudes and the stellar radius of 21.0, 17.6 and 0.13 R_Sun, resp., Therefore, it is only necessary to estimate S_K. For a V - K of 3.4, Bailey's approximation gives S_K = 4.29 and Berriman's gives 4.21, resulting in distances of 597 and 619 pc, resp. For a distance of >1 kpc, the S_K would need to be as low 3.15 or the K magnitude >18.0. Both of which are highly discrepant from your observations and previous analyses. Therefore, the distance to the ASKAP J1745-5051 is unlikely to be >1 kpc. The authors are encouraged to use the 1 kpc upper limit in the paper.

As a result, the discussions quoting a distance of 9.1 kpc should be changed to 1 kpc, namely lines 213 and 446, and extended data figures 3 and 4 should be adjusted to show this change. The discussion about the Bailer-Jones photogeometric distance is also clearly incorrect based on this discussion and should be removed.

Note that a measurement of the K band magnitude should settle this issue.

>>> We note on line 622 the caveats about how our blackbody fitting may be impacted by contamination from accretion structure, the nearby star (Gaia 1488), or possibly both. We have added a comment to this line noting that such contamination, along with the poorly constrained blackbody fit, may imply higher than reasonable brightness at longer wavelengths; and lowered upper x-limit on the blackbody figure to limit extrapolation beyond wavelengths that we observe. Since we do not explicitly rely upon the 17.5 mag K-band detection, we defer a measurement of the K band magnitude to future work. We retain the conservative distance range of 0.4–9.1 kpc to avoid making further assumptions about the optical emission, but we have added a comment in the Gaia Methods section noting that the true distance is likely closer to the parallax value.

2. The paper adequately addresses the issue of gyrosynchrotron emission.

Minor issues:

1. Ln 221: The paper emphasizes that the radio luminosity from J1745-5051 is ~100 greater than 99% of CVs, but almost all of those observations are at higher frequencies and are caused by the electron cyclotron maser instability (ECMI). Whereas, the radio emission from J1745-5051 is unlikely to be caused by relativistic ECMI (see Comment 1). Therefore, what is the point of this statement and how does it add to our understanding of this source?

>>> We demonstrate (lines 749-788) that ECMI is the most likely driver of the observed emission from ASKAP J1745.

2. Ln 735: This section should list the observed dispersion measure or its upper limit.

>>> We do not measure a dispersion measure or upper limit. The intention of this section is to search for single pulses that are dispersed by up to twice the maximum Galactic contribution for dispersion.

3. Ln 785: The paper should include an estimate of the radio luminosity from the low density plasma (< 10^11 cm^-3) to support their assertion that ECMI from such a low density plasma can produce the observed luminosity.

>>> Lower density plasma is necessary to produce ECMI; e.g. Treumman 2006 (https://doi.org/10.1007/s00159-006-0001-y). The simulated dynamic spectra we present come from a geometric model of the pulse visibility at different times/frequencies as generated by relativistic ECMI, ignoring several physical parameters of the plasma environment which are unconstrained by the available observations. Full characterisation of the emission (including luminosity) will require detailed physical modelling of the plasma environment as well as further development of current relativistic ECMI theory.

Comments:

1. Ln 282 & 769: Note that paper [45] proposing relativistic ECMI is almost certainly incorrect, because ECMI requires a non-thermal velocity distribution that is truncated at high energies. Therefore, it is unlikely that a relativistic population of electrons can be generated with such characteristics as the synchrotron timescale is much shorter than the ECMI timescale.

>>> Relativistic ECMI has been proposed as a feasible mechanism for LPTs; e.g. Ferrario 2026 (https://ui.adsabs.harvard.edu/abs/2026MNRAS.546ag146F/abstract). We leave more detailed considerations of the precise velocity distribution to future work.
